# An improved genome assembly for *Larimichthys crocea* reveals hepcidin gene expansion with diversified regulation and function

**DOI:** 10.1038/s42003-018-0207-3

**Published:** 2018-11-16

**Authors:** Yinnan Mu, Jieying Huo, Yanyun Guan, Dingding Fan, Xiaoqiang Xiao, Jingguang Wei, Qiuhua Li, Pengfei Mu, Jingqun Ao, Xinhua Chen

**Affiliations:** 1 Institute of Oceanology, College of Animal Sciences, Fujian Agriculture and Forestry University, 350002 Fuzhou, China; 2 Key Laboratory of Marine Biogenetic Resources, Third Institute of Oceanography, State Oceanic Administration, 361005 Xiamen, China; 3 EasyATCG L.L.C, Shenzhen, China; 4 College of Marine Sciences, South China Agricultural University, Guangzhou, China; 5 Laboratory for Marine Biology and Biotechnology, Qingdao National Laboratory for Marine Science and Technology, Qingdao, China

## Abstract

*Larimichthys crocea* (large yellow croaker) is a type of perciform fish well known for its peculiar physiological properties and economic value. Here, we constructed an improved version of the *L. crocea* genome assembly, which contained 26,100 protein-coding genes. Twenty-four pseudo-chromosomes of *L. crocea* were also reconstructed, comprising 90% of the genome assembly. This improved assembly revealed several expansions in gene families associated with olfactory detection, detoxification, and innate immunity. Specifically, six hepcidin genes (LcHamps) were identified in *L. crocea*, possibly resulting from lineage-specific gene duplication. All LcHamps possessed similar genomic structures and functional domains, but varied substantially with respect to expression pattern, transcriptional regulation, and biological function. LcHamp1 was associated specifically with iron metabolism, while LcHamp2s were functionally diverse, involving in antibacterial activity, antiviral activity, and regulation of intracellular iron metabolism. This functional diversity among gene copies may have allowed *L. crocea* to adapt to diverse environmental conditions.

## Abstract (web summary)

Yinnan Mu and colleagues present an improved yellow croaker genome, constructed using a hybrid assembly method incorporating long-reads and short-reads. With their improved assembly, the authors found several gene family expansions associated with olfactory detection, detoxification, and innate immunity.

## Introduction

The large yellow croaker (*Larimichthys crocea*), a temperate-water migratory fish belonging to the order Perciformes and the family Sciaenidae, is one of the most economically important marine fish in China and East Asia^1^. In addition to its economic value, *L. crocea* is a unique model species with some peculiar traits: it produces loud sounds, has a well-developed olfactory system, and is sensitive to various environmental stressors, such as hypoxia and chemical pollution^2^. The transcriptomic and proteomic responses of *L. crocea* to various stimuli, including pathogens, hypoxia, and high temperature have been investigated extensively to determine the specific defense mechanisms it uses to handle physiological responses^3–9^. The whole genome of *L. crocea* was previously sequenced with Ilumina HiSeq 2000 system^2,10^. However, this approach is limited by the short read lengths, inherent sequencing biases, and non-random sequencing errors, which result in highly fragmented draft genome assemblies that may overlook biologically meaningful sequences^11^. Single molecule real-time (SMRT) sequencing, developed by Pacific BioSciences (USA), offers an alternative approach that overcomes these limitations^12^. Indeed, the combination of Ilumina and SMRT sequencing technologies is an economical and effective strategy for the assembly of highly polymorphic genomes.

Teleost fish, living in complex aquatic environments, have evolved various genetic mechanisms to adapt to ever-changing environments, including gene expansion and positive selection^13^. Gene expansion is a remarkably flexible mechanism that promotes the emergence of evolutionary novelties and functional diversification^14^. Of the expanded genes, hepcidin, a small amphipathic antimicrobial peptide^14^, is currently known only in vertebrates. All mammals possess a single hepcidin gene except mice (*Mus musculus*), which possess two^15^. Two hepcidins were also reported in an amphibian (Western clawed frog, *Xenopus tropicalis*)^16^, while a single hepcidin gene was identified in birds^17^. Interestingly, some teleost fish possess several hepcidin genes, including the orange-spotted grouper *Epinephelus coioides* (three copies)^18,19^, the tilapia *Oreochromis mossambicus* (three copies)^20^, the rockbream *Oplegnathus fasciatus* (four copies)^21^, and seabass *Dicentrarchus labrax* (five copies)^22^. Fish hepcidins are phylogenetically classified into two groups: the Hamp1-type, with a single isoform that shares a considerable degree of homology with its mammalian counterparts, and the Hamp2-type, with various diverse isoforms unique to fish^22^. In mammals, hepcidins primarily regulate iron metabolism, but in fish they are predominantly antimicrobial acting against gram-negative bacteria, gram-positive bacteria, and fungi^22–24^. Unexpectedly, the hepcidins of tilapia^25^, grouper^26^, and spotted scat (*Scatophagus argus*)^23^ also exhibit antiviral properties. Induction of zebrafish (*Danio rerio*) hepcidin decreases iron circulation, thereby increasing the concentration of intracellular iron^27^. Conversely, in the turbot (*Scophthalmus maximus*), hepcidin induction reduced intracellular chelatable iron in kidney cells^28^. Because teleost fish have evolved more hepcidin genes than mammals, it is essential to determine the functional differences of these distinct hepcidin genes.

In this study, we constructed a markedly improved genome assembly and chromosome map of *L. crocea*. We used this genome to identify multiple previously unrecognized expansions in genes coding olfactory receptors, cytochrome P450 proteins, pattern recognition receptors, chemokines and antimicrobial peptides. These encoded proteins were associated with olfactory detection, elimination of foreign chemicals, and innate immunity. We identified six unique hepcidin (LcHamp) genes in *L. crocea* and investigated their tissue distributions, promoter characteristics, and functions (as antibacterial/antiviral agents and/or as regulators of iron metabolism). These data afforded new insights into the adaptive mechanisms used by this species to thrive in diverse environmental conditions and provided a framework for the genetic improvement of *L. crocea*.

## Results

### Comparison of two versions of *L. crocea* genome assembly

**Fig. 1:**
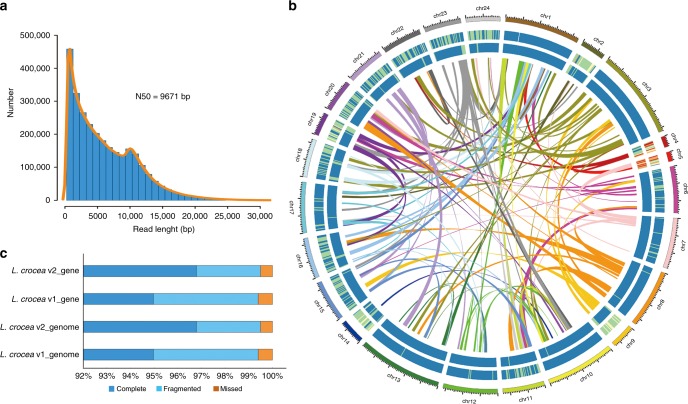
Genome assembly of *Larimichthys crocea*. **a** Length distribution of single long reads. **b** Comparison between the first and second version of the *L. crocea* genome, showing the portions of the genomes that are complete (blue), fragmented (light blue), or missed (brown), as determined by benchmarking universal single-copy orthologs (BUSCO) analysis. **c** Circos atlas representation of the chromosome assembly information. The outermost circle shows the 24 pseudo-chromosomes of *L. crocea*. The next circle shows the gene density within a 500 kb window and the innermost circle shows the density of transposon elements within a 500 kb window. Links display homologous regions in the *L. crocea* genome along its 24 pseudo-chromosomes

The genome size of *L. crocea* was about 669.78 Mb, with a high level of genome heterozygosity (1.06%; Supplementary Table 1 and Supplementary Fig. 1). To obtain a higher-quality genome assembly, we used a hybrid assembly method: a 25-fold coverage of the long molecule sequences from SMRT, with an N50 of 9.67 kb and a longest read length of 133.08 kb (Fig. 1a and Supplementary Table 2), and a 563-fold coverage of short read sequences from Illumina (Supplementary Tables 3 and 4). The overall assembly statistics of our new genome version (*L. crocea* v2.0) were dramatically better than the original version: scaffold N50 increased from 1.03 Mb to 6.55 Mb, contig N50 increased from 63.11 kb to 282.69 kb, number of contig N90 decreased from 11,390^2^ to 3097 (Supplementary Tables 5 and 6). Importantly, more than 99.88% of the high-quality reads could be mapped to the improved assembly, which was higher than that of the previous genome version (95.63%)^2^. Moreover, all 248 highly conserved genes tested and 99.6% of orthologous genes of Actinopterygii could be mapped to the improved assembly (Fig. 1b and Supplementary Table 7). Finally, 24 pseudo-chromosomes of *L. crocea* were reassembled using 4778 high-quality single-nucleotide polymorphisms, representing 90% of the genome assembly (604.4 Mb; Fig. 1c and Supplementary Table 8). These data indicated that the new versions of the *L. crocea* genome assembly and chromosome map were substantially better than the original versions.

### Comparison of gene family between *L. crocea* and other teleost fish

Using EvidenceModeler, we predicted 26,100 genes, of which 93.75% were supported by at least two methods (Supplementary Table 9). The ratio of genes with complete open reading frames increased from 47.02% in the original genome assembly to 93.66% in our improved assembly (Supplementary Table 9), while the proportion of the fragmented genes decreased from 4.4% to 2.7% (Fig. 1b). Over 97.39% of all inferred proteins matched entries in at least one database, including GenBank, InterPro, Swissprot, Kyoto Encyclopedia of Genes and Genomes, and Gene ontology databases. Moreover, 17,414 gene families of 19,627 orthologous gene families from *Danio rerio*, *Gasterosteus aculeatus*, *Takifugu rubripes* were identified in the *L. crocea* v2.0 genome, including 142 novel gene families that were not found in the original genome assembly (Supplementary Table 10). We also identified 68 novel expanded gene families (*P* < 0.05, Supplementary Table 11), some of which are associated with olfactory detection (olfactory receptor genes: OR2B11 and OR4C5) and detoxification (cytochrome P450 genes: CYP2J2, CYP2J6, and CYP2K1). We also identified expansions in several immune-relevant genes, including macrophage mannose receptors (MRC1 and MRC2), chemokines (CCL2, CCL3, and CCL5) and antimicrobial peptide (hepcidins). These gene expansions probably enhance the innate immunity of *L. crocea* against pathogen invasion.

### Molecular characterization of *L. crocea* hepcidins

**Fig. 2:**
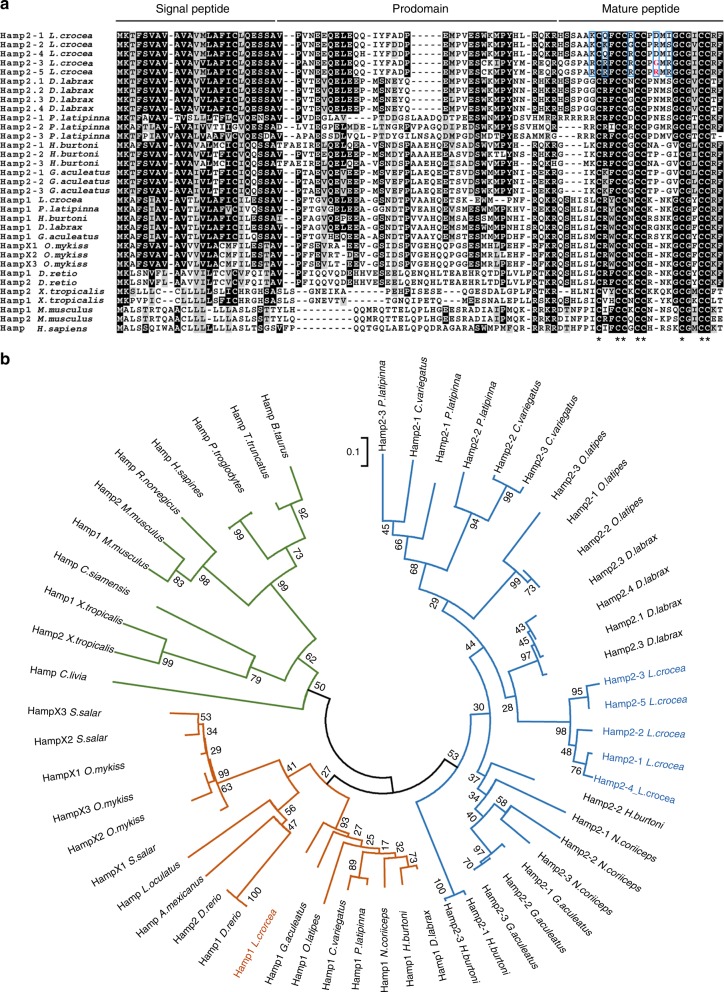
Hepcidin sequence alignment and phylogenetic analysis. **a** Alignment of multiple hepcidin amino acid sequences from *L. crocea* and other vertebrates. Sequence alignment was obtained using the CLUSTAL W2. Conserved residues are shaded with BOXSHADE v3.21. The eight highly conserved cysteine residues are indicated with asterisks. The only different amino acid (arginine75 in HAMP2-5/glycine75 in HAMP2-3) between LcHamp2-3 and LcHamp2-5 mature peptides was indicated by red font. The variant arginines in LcHamp mature peptides were shown with blue boxes. **b** Phylogenetic tree of hepcidins based on deduced amino acid sequences. Deduced amino acid sequences of hepcidins were aligned, and the tree was constructed with the Neighbor-Joining method using the MEGA v6. The tree is bootstrapped 10,000 times, and the bootstrap values of the major branches are shown as percentages. The mammalian, avian, reptile, and amphibian Hamps are indicated with green lines. Fish Hamp1s and Hamp2s are indicated with brown and blue lines, respectively

Importantly, our new assembly of *L. crocea* revealed six distinct hepcidin genes on chromosome 13 (Supplementary Fig. 2), named *Lchamp1*, *Lchamp2-1*, *Lchamp2-2*, *Lchamp2-3*, *Lchamp2-4*, and *Lchamp2-5*. To our knowledge, this is a higher number of copies of the hepcidin gene than has been seen in other species (Supplementary Fig. 3). Each *Lchamp* gene was comprised of three exons and two introns; this structure is highly conserved across species (Supplementary Fig. 4). As in mammals, the LcHamp1 and LcHamp2 prepropeptides both included three primary domains: a hydrophobic signal peptide, a prodomain, and a mature peptide (Fig. 2a and Supplementary Fig. 5). Alignment of the *L. crocea* hepcidins with those of other vertebrate species indicated that the mature peptide region was highly conserved, particularly the 8 cysteine residues (Fig. 2a). LcHamp2-1 and LcHamp2-4 had identical mature peptide sequences.

The phylogenetic analysis indicated two hepcidin subgroups in fish: the Hamp1-type and the Hamp2-type (Fig. 2b). Hamp1s were present in all fish species we analyzed; Hamp2s were primarily identified in fish, specifically in the Percomorphaceae and the Ovalentaria (Fig. 2b and Supplementary Fig. 6). In our phylogeny, LcHamp1 fell in the Hamp1 clade, while other *L. crocea* hepcidins clustered with the Hamp2s. We used maximum likelihood methods to explore the selective pressures acting on all Osteichthyes hepcidin genes. Our likelihood ratio tests indicated that model M3 (three distinct rates of nucleotide substitution across all sites) fit our data better than model M0 (a single rate of nucleotide substitution across all sites; *P* < 1e-9), suggesting that rate of nucleotide substitution was heterogeneous among different amino acid sites (Supplementary Table 12). Further likelihood ratio tests suggested that the positive selection model (M2a) fit our data significantly better than the nearly neutral model (M1a) (*P* = 0.000007866), and that positive selection had acted on the codons (M8 vs. M7; *P* = 0.000003608 for M8 vs. M7).

### Hepcidin expression in *L. crocea* tissues

**Fig. 3:**
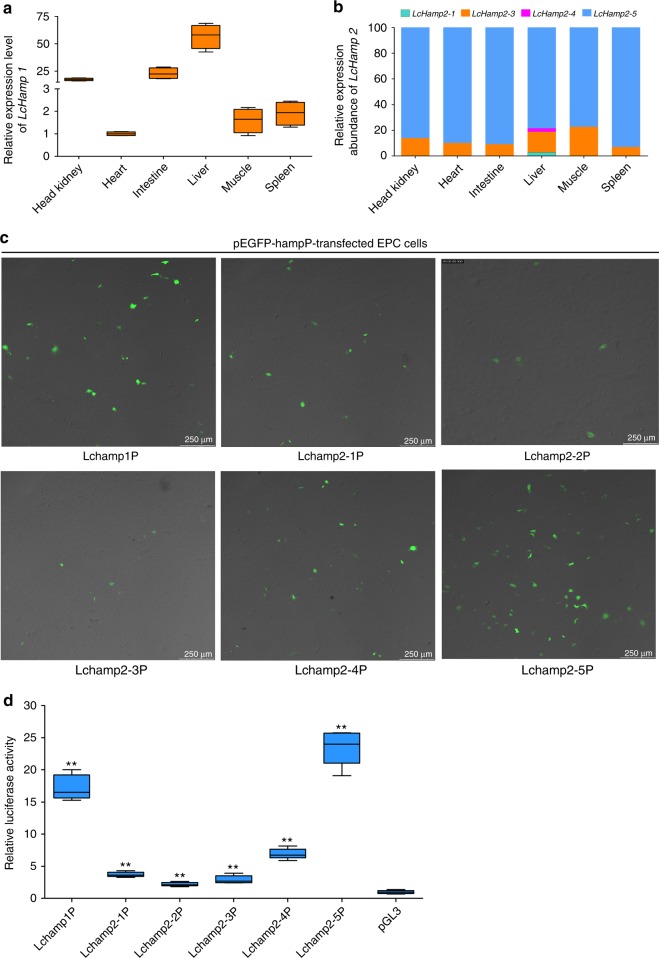
Tissue distribution and promoter activity of *Larimichthys crocea* hepcidin genes. **a** The relative expression levels of *LcHamp1* in various tissues, normalized to β-actin using the 2^−ΔΔCT^ method and expressed relative to *LcHamp1* expression in the heart. **b** Relative expression abundance of *LcHamp2* isoforms across the different tissues of *L. crocea*. The relative expression abundance of each *LcHamp2* isoform was determined by counting the number of clones of each *LcHamp2* isoform out of 200 clones randomly selected from each of the six tissues tested. **c** Fluorescent microscope images of epithelioma *papulosum cyprini* cells transfected with recombinant plasmids containing the promoters of each *Larimichthys crocea* hepcidin gene. **d** The transcriptional activity of the *Larimichthys crocea* hepcidin promoters. Firefly luciferase activity was normalized to Renilla luciferase activity, and the relative luciferase activity was expressed as the ratio of normalized luciferase activity in cells transfected with pGL3-LchampP plasmid to the normalized luciferase activity in control cells transfected with the pGL3-Basic plasmid. All data were collected in three independent experiments with three replicates per experiment. Error bars indicate the standard error of the mean of the three independent experiments. ^*^P < 0.05; ^**^P < 0.01

We measured the expression of the hepcidin genes in various tissues of *L. crocea* (the head kidney, heart, intestine, liver, muscle, and spleen). *LcHamp1* mRNA expression was detected in all tissues tested but was most highly expressed in the liver (Fig. 3a). Due to the very high sequence similarity among *LcHamp2* isoforms, we designed a pair of conserved primers (Hamp2-F1 and Hamp2-R1; Supplementary Table 13) to simultaneously amplify all *LcHamp2* isoforms. We measured the relative expression of each *LcHamp2* isoform by calculating the number of clones of each *LcHamp2* isoform out of 200 randomly selected clones from each tissue. Both *LcHamp2-3* and *LcHamp2-5* were constitutively and highly expressed in all six evaluated tissues; *LcHamp2-5* was more highly expressed than any other *LcHamp2* isoform (Fig. 3b). *LcHamp2-1* and *LcHamp2-4* were only expressed in the liver, and *LcHamp2-2* expression was not detected in any tissue. Therefore, the different *LcHamp2* isoforms had dramatically different expression patterns.

### Characterization of the *L. crocea* hepcidin promoters

Gene expression level is closely related to the basal transcriptional activity of the gene promoter^29^. To explore the molecular basis for the varied expression patterns of the six *L. crocea* hepcidin genes, we cloned the promoter region of each gene (Supplementary Fig. 7) and measured the transcriptional activity of each promoter using recombinant reporter gene plasmids, pEGFP-LchampPs and pGL3-LchampPs in epithelioma papulosum cyprini (EPC) cells. All *Lchamp* promoters drove enhanced green fluorescent protein (EGFP) reporter gene expression (Fig. 3c) and initiated luciferase reporter gene transcription in EPC cells (Fig. 3d). Transcriptional activity was highest in the EPC cells transfected with pGL3-Lchamp2-5P1, while lowest in the EPC cells transfected with pGL3-Lchamp2-2P1.

**Fig. 4:**
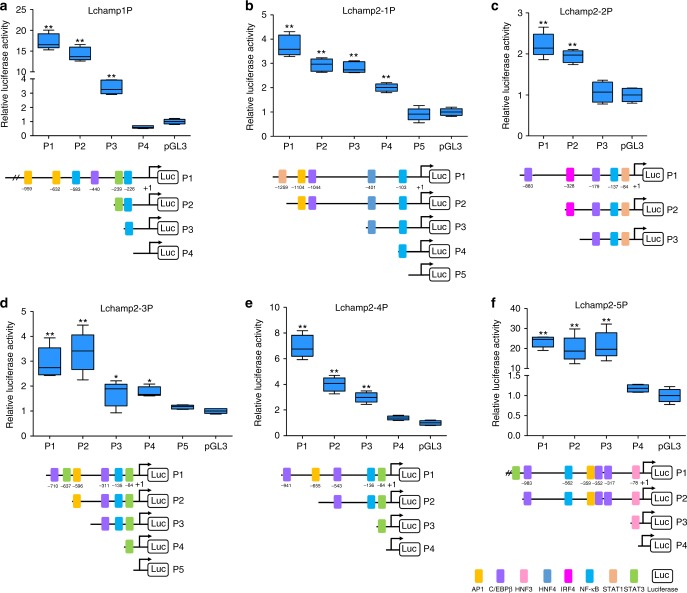
The structure and transcriptional activity of the *Larimichthys crocea* hepcidin promoters. Relative luciferase activity and schematic structural representation of the promoters of (**a**) *Lchamp1*, (**b**) *Lchamp2-1*, (**c**) *Lchamp2-2*, (**d**) *Lchamp2-3*, (**e**) *Lchamp2-4*, and (**f**) *Lchamp2-5*. Several deletion constructs for each *L. crocea* hepcidin promoter were constructed based on predicted transcription factor binding sites. Epithelioma papulosum cyprini (EPC) cells were seeded in 96-well plates (5 × 10^4^/well) overnight and co-transfected with 100 ng of pGL3-LchampP plasmid and 2 ng of pRL-TK using the FuGENE HD transfection reagent. After 48 h of transfection, luciferase activity in the EPC cells was measured. Firefly luciferase activity was normalized to Renilla luciferase activity, and the relative luciferase activity was expressed as the ratio of normalized luciferase activity in cells transfected with pGL3-LchampP plasmid to the normalized luciferase activity in control cells transfected with the pGL3-Basic plasmid. All data were collected in three independent experiments with three replicates per experiment. Error bars indicate the standard error of the mean of the three independent experiments. ^*^P < 0.05; ^**^P < 0.01

We then searched for the putative transcription factor binding sites (i.e., STATs, C/EBPs, AP1, NF-κB, and HNFs) on *Lchamp* promoters with MatInspector^30^. All *Lchamp* promoters contained most of the transcription factor binding sites, except that *Lchamp2-2* promoter lacked the AP1 binding site, *Lchamp1*, *Lchamp2-3,* and *Lchamp2-4* promoters lacked the HNF binding site, and the IRF4 binding site was only found in *Lchamp2-2* promoter (Supplementary Fig. 7). Based on these results, we constructed several truncated mutants of *Lchamp* promoters and measured transcriptional activity of each mutant. Deletion of the NF-κB binding site greatly reduced the basal activities of promoters of *Lchamp1* and *Lchamp2-1*, suggesting that the NF-κB binding site was important for induction of these two genes (Fig. 4a, b). Similarly, the IRF4 and HNF3 binding sites were required for basal activities of the promoters of *Lchamp2-2* and *Lchamp2-5*, respectively, while the STAT3 binding site was required for the basal activities of the promoters of *Lchamp2-3* and *Lchamp2-4* (Fig. 4c–f).

### Activity of synthetic mature peptides of *L. crocea* hepcidins

To clarify the functional differences among the distinct *Lchamp* isoforms, we synthesized the five types of mature LcHamp peptides: HAMP1, HAMP2-1/4, HAMP2-2, HAMP2-3, and HAMP2-5. We evaluated the antimicrobial activities of these peptides using minimum inhibitory concentration assays. HAMP2-3 and HAMP2-5 had an antimicrobial effect on five gram-negative bacteria (*Aeromonas hydrophila*, *Escherichia coli*, *Vibrio alginolyticus*, *Vibrio harvryi*, and *Vibrio parahaemloyticus*) and three gram-positive bacteria (*Bacillus subtilis*, *Bacillus amyloliquefaciens*, and *Staphylococcus aureus*; Table 1). HAMP2-5 was more potent, with minimum inhibitory concentrations for the bacterial species tested ranging from 0.25 µM to 1 µM. However, HAMP1, HAMP2-1/4, and HAMP2-2 showed no antibacterial activity in response to any tested bacteria even at concentrations as high as 300 µM.

**Table 1:** Minimal inhibitory concentration of the synthetic *Larimichthys crocea* hepcidins

Microorganisms	MIC (µM)	
	HAMP2-3	HAMP2-5
Gram-negative bacteria		
* Aeromonas hydrophila*	16	1
* Escherichia coli*	16	0.5
* Vibrio alginolyticus*	16	0.25
* Vibrio harveyi*	16	0.5
* Vibrio parahaemloyticus*	16	0.5
Gram-positive bacteria		
* Bacillus subtilis*	4	0.25
* Bacillus amyloliquefaciens*	8	1
* Staphylococcus aureus*	4	0.5

**Fig. 5:**
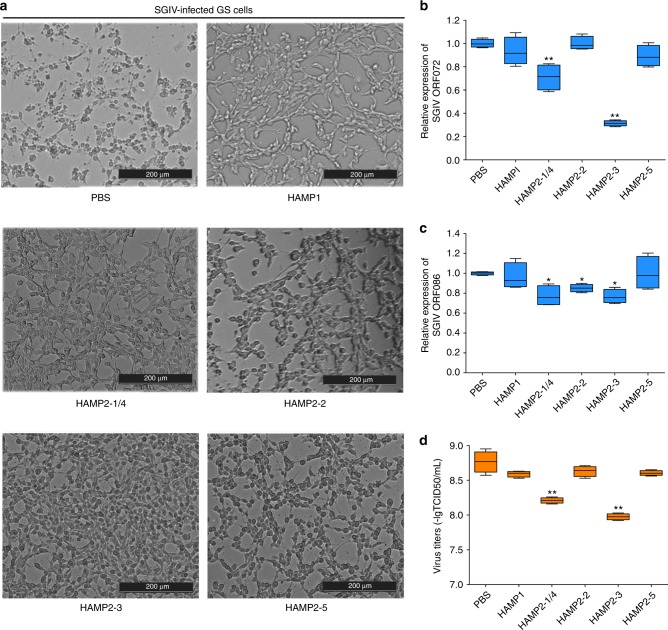
Antiviral activity of synthetic mature peptides of *Larimichthys crocea* hepcidin. **a** Phase micropictographs of cytopathic effects induced by Singapore grouper iridovirus (SGIV) infection in grouper spleen cells, pretreated with a synthetic mature peptide of *L. crocea* hepcidin (HAMP1, HAMP2-1/4, HAMP2-2, HAMP2-3, or HAMP2-5) or with phosphate buffered saline (control). At 48 h post-infection, the expression levels of the SGIV genes (**b**) *ORF072* and (**c**) *ORF086* were measured with real-time PCR and normalized to *Ecβ-actin*. **d** SGIV titers in grouper spleen cells after treatment with hepcidin mature peptides. HAMP1, HAMP2-2, and HAMP2-5 exhibited no antiviral activity. Error bars represent the standard error of the mean of three independent experiments. ^*^P < 0.05; ^**^P < 0.01

We measured the antiviral activity of synthetic *L. crocea* hepcidins in a viral infection model, where grouper spleen cells were infected with the Singapore grouper iridovirus (SGIV). HAMP2-1/4 and HAMP2-3 exhibited antiviral activity against SGIV infection, based on less cytopathic effect, decreased viral gene expression (*ORF072* and *ORF086)*, and reduced viral titer 50% tissue culture infectious dose in HAMP2-1/4- and HAMP2-3-treated cells (Fig. 5). However, HAMP1, HAMP2-2, and HAMP2-5 exhibited no identifiable antiviral activity.

**Fig. 6:**
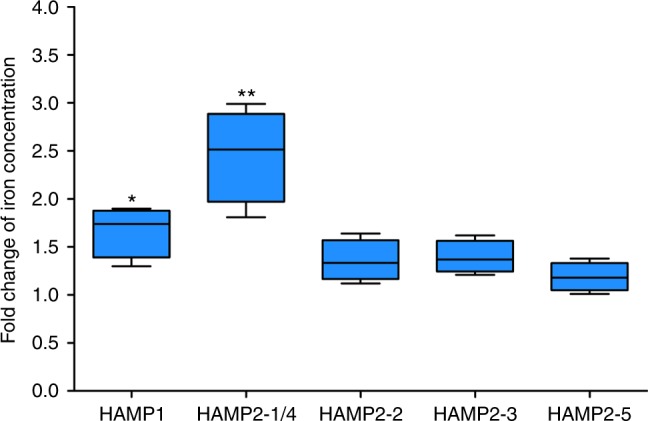
Change in intracellular iron concentration in macrophages treated with synthetic mature peptides of *Larimichthys crocea* hepcidins. Variations in intracellular iron of macrophages were detected with Inductively Coupled Plasma Mass Spectrometry after treatment with a single mature hepcidin peptide. Fold change was determined by comparing the iron concentration of peptide-treated cells with that in control cells treated with phosphate buffered saline. Error bars represent the standard error of the mean of three independent experiments. ^*^P < 0.05; ^**^P < 0.01

We then tested the regulatory effect of *L. crocea* hepcidins on iron metabolism. The increases in iron content were observed in HAMP1- and HAMP2-1/4-treated macrophages from *L. crocea* head kidney (*P* < 0.05, Fig. 6), suggesting that LcHamp1 and LcHamp2-1/4 may play a role in regulation of iron metabolism.

## Discussion

As genome sequencing technology rapidly develops, the genome assemblies of many species have been updated^31–33^. These more complete sequences have allowed the identification of previously unrecognized, biologically meaningful sequences. Here, we constructed an improved genome assembly for *L. crocea*, with a 4.5-fold increase in contig N50, a 6.4-fold increase in scaffold N50, and 2-fold increase in the proportion of complete open reading frames, as compared to the previous version of the *L. crocea* genome^2^ (Supplementary Table 6). Using this updated version of the genome, we reconstructed 24 pseudo-chromosomes of *L. crocea*, comprising 90% of the genome assembly (Fig. 1c and Supplementary Table 6). Relatively complete genomes and chromosome maps are critical for locating genes related to quantitative traits, such as those associated with disease resistance, growth, and sex determination. Such information will facilitate species improvement of *L. crocea* through selective breeding or genetic modification. The improved assembly also revealed multiple expansions in gene families (Supplementary Table 11). Expansion of olfactory receptor genes, OR2B11 and OR4C5, further supports our previous speculation that olfactory receptor gene expansions have contributed to the olfactory sensitivity of *L. crocea* and facilitated its feeding and migration^2^. Also expanded were the cytochrome P450 genes (CYP2J2, CYP2J6, and CYP2K1), which are involved in the metabolism of endogenous and exogenous chemicals^34^. CYP2J2 is a prominent enzyme responsible for metabolizing endogenous polyunsaturated fatty acids to signaling molecules. CYP2K1 is the major hepatic P450 form catalyzing the bioactivation of carcinogen aflatoxin B1. The expansion of these cytochrome P450 genes may help *L. crocea* cope with increasing chemical exposures. We also identified expansions in macrophage mannose receptors, CC chemokines and hepcidin genes. Macrophage mannose receptors bind high-mannose containing structures on the surface of potentially pathogenic bacteria, viruses, and fungi, thus facilitating the neutralization of these pathogens by phagocytic engulfment^35^. CCL2, CCL3, and CCL5 belonging to inflammatory chemokines, are produced in high concentrations during infection or injury and determine the migration of inflammatory leukocytes into the damaged area^36–40^. All these gene expansions therefore provide new insights into the mechanisms underlying the adaptation of this species to diverse environmental conditions.

Notably, we identified six hepcidin genes in our new assembly of the *L. crocea* genome, more than have currently been reported in other fish, to our knowledge (Supplementary Fig. 3). All LcHamp proteins possessed a consistent arrangement of 8 conserved cysteines (Fig. 2a) which are believed to be essential for hepcidin activity^41^. However, in our phylogenetic analysis, the LcHamp proteins fell in different clusters (Fig. 2b). LcHamp1 clustered with Hamp1s, which have been identified in all fish species analyzed. In contrast, LcHamp2 isoforms clustered with Hamp2s that have only been identified in fish belonging to Percomorphaceae and Ovalentaria (Fig. 2b and Supplementary Fig. 6), suggesting that the multiple Hamp2 genes found in fish may have resulted from lineage-specific gene duplication. We also identified signals of positive selection among the protein-coding sites of fish hepcidin genes, accompanied by variations in charge and polarity (Supplementary Table 12). The expansion of the hepcidins, and the positive selection pressure on these genes, might be explained by adaptive evolution in response to diverse ecological conditions, including pathogens, hypoxia, and excess iron.

In humans, hepcidin is primarily expressed in liver and is induced by various stimuli (e.g., cytokines, plasma iron, and hypoxia) through SMAD and JAK-STAT signaling pathways^42,43^. Fish hepcidins are usually highly expressed in the liver or kidney^22–24^, but the mechanisms regulating their expression remain unclear. Here, *LcHamp1* was highly expressed in the liver (Fig. 3a), similar to human hepcidin and other fish *Hamp1*^22,43^. *LcHamp2* isoforms had different expression abundance in various tissues (Fig. 3b). *LcHamp2-5* was the most predominantly expressed of all *LcHamp2* isoforms, while *LcHamp2-2* was not detected in all examined tissues, which are consistent with the basal transcriptional activities of their promoters (Fig. 3c, d). Meanwhile, LcHamp2-2 showed no antibacterial activity, antiviral activity or regulatory effect on iron metabolism (Table 1 and Figs. 5, 6). The lack of detectable tissue expression and functional activity suggests that LcHamp2-2 may have undergone a functional diminishment during the course of evolution. Additionally, the critical transcription factor binding site for each *Lchamp* promoter varied: *Lchamp1* and *Lchamp2-1* required the NF-κB binding site; *Lchamp2-2* required the IRF4 binding site; *Lchamp2-3* and *Lchamp2-4* required the STAT3 binding site; and *Lchamp2-5* required the HNF3 binding site (Fig. 4). In mice, the NF-κB and STAT3 have been confirmed to regulate hepcidin expression during inflammatory conditions^44,45^. Surprisingly, IRF4, which is well-known to control Th2, Th17, and T regulatory cell specification^46^, was shown to also regulate *LcHamp2-2* expression. These differences in tissue expression, promoter activity, and transcription factor binding sites suggest that the expression of *LcHamp*s may be regulated by different transcription factors responding to highly dissimilar stimuli.

In mammals, a single hepcidin acts both as a bactericidal peptide and as a homeostatic regulator of iron metabolism^27^. In contrast, many fish possess multiple copies of hepcidin genes (Fig. 2b). Previous reports speculated that Hamp1 acted solely as a regulator of iron metabolism, while Hamp2 primarily performed an antimicrobial role^22^. Here, the mature peptides of LcHamp2-3 and LcHamp2-5 inhibited the growth of various gram-positive and gram-negative bacteria (Table 1). Interestingly, the activity of HAMP2-5 was much more potent than that of HAMP2-3, although they differ by only one amino acid (arginine^75^ in HAMP2-5/glycine^75^ in HAMP2-3; Fig. 2a), indicating that the arginine^75^ in HAMP2-5 is important for its antibacterial activity. HAMP2-1/4 and HAMP2-2, both with four fewer arginines (R^65^, R^67^, R^77^, and R^71^/R^75^) than HAMP2-5, displayed no antimicrobial activity, suggesting that the arginine residues at these positions in the mature peptide of hepcidins may be critical for their antimicrobial properties. The mature LcHamp1 peptide did not have any antimicrobial effects, but markedly increased iron concentration in macrophages (Fig. 6), supporting the previous proposal that Hamp1 is a major regulator of iron metabolism^22,47,48^. HAMP2-1/4 treatment also could increase intracellular iron of macrophages, indicating that Hamp2s regulated iron metabolism as well, differing from the previous observations^22,23^. Finally, HAMP2-1/4 and HAMP2-3 markedly inhibited SGIV replication (Fig. 5). These results clearly revealed the functional activity of each LcHamp isoform, thus providing the solid basis for the targeted use of these antimicrobial peptides in aquaculture. Teleost fish live in complex aquatic environments, particularly threatened by various environmental stressors. The gene expansion and functional divergence of hepcidins might have been important in the adaptation of fish to diverse ecological conditions.

In the present study, we generated an improved genome assembly and a high-quality chromosome map for *L. crocea* and identified multiple expansions in genes associated with olfactory detection, elimination of foreign chemicals, and innate immunity. Of these expanded genes, the six hepcidin isoforms had similar molecular characteristics, but possessed unique expression patterns, transcriptional regulation, and functions. These results revealed the gene expansions and functional divergences of the hepcidin in teleost fish. Finally, our study also serves as a framework for mapping quantitative trait loci of economically important traits, thus facilitating genetic improvement of *L. crocea*.

## Methods

### Preparation of fish tissue samples

Large yellow croaker fish (mean mass: 103 ± 21.9 g; mean length: 21 ± 1.3 cm) were purchased from a mari-culture farm in Ningde, Fujian, China. Fish were maintained in tanks supplied with 25 °C flow-through seawater. After seven days of acclimation, the fish were killed and tissues were used as described below. The studies were carried out in strict accordance with the Regulations of the Administration of Affairs Concerning Experimental Animals established by the Fujian Provincial Department of Science and Technology. All surgery was performed under Tricaine-S anesthesia, and all efforts were made to minimize suffering.

### Cell lines and virus

EPC cells (obtained from the China Center for Type Culture Collection, Wuhan, China) were derived from fathead minnow, *Pimephales promelas*, and cultured at 25 °C in L-15 medium supplemented with 10% fetal bovine serum^49^. Grouper spleen cells obtained from the spleen of orange-spotted grouper, were maintained in L-15 medium supplemented with 10% fetal bovine serum at 25 °C. SGIV was propagated in grouper spleen cells as previously described^50^, and the virus stock was stored at −80 °C until use. Macrophages were isolated from the head kidney tissues of *L. crocea*, as previously described^39^, and cultured at 28 °C in L-15 medium.

### Genome sequencing and assembly

We extracted genomic DNA from muscle of one female wild large yellow croaker (FUFA-1) using the phenol-chloroform method for SMRT sequencing. For the PacBio Sequel system, extracted DNA was sheared into 20 kb fragments using G-tubes (Covaris, USA), then enzymatically repaired and converted into 20 kb SMRT bell template libraries as recommended by Pacific Biosciences. Libraries were size-selected with a lower cutoff of 7 kb using BluePippin (Sage Science, USA). Sequencing was performed on the PacBio Sequel system using the Sequel Sequencing Kit 2.0. In total, three SMRT cells were sequenced, producing about 2.81 million of reads containing 16.70 giga bases.

Illumina read libraries were obtained from the previously published draft *L. crocea* genome (GenBank accession no. JRPU00000000)^2^. High quality reads with about 50-fold coverage were used to evaluate genome size and calculate kmer curve by using SOAPdenovo2^51^. Jellyfish v2.2.6^52^, set to count all kmers (-C -m 21), and GenomeScope (http://qb.cshl.edu/genomescope/)^53^ were used to calculate genome heterozygosity. We reassembled the *L. crocea* genome using the Platanus assembler v1.2.1^54^. The short reads (170–500 bp) were used to build contig with default parameters, and scaffolds were oriented by adding mated pair sequencing reads (2–40 kb). Gaps in the initial assembly were gradually filled with reads from the short-insert libraries using GapCloser^51^, and long sequences from BAC libraries using GMCloser v1.6^55^. Finally, the remaining gaps were filled with PacBio reads using PBSuite v15.8.24^56^. The genome assembly was polished with Quiver (https://github.com/PacificBiosciences/GenomicConsensus) and Pilon v1.22^57^ using high quality reads from the short-insert libraries.

Assembly complexity was measured by aligning high quality short reads (56-fold coverage) to the genome assembly using Burrows-Wheeler Aligner^58^ with default parameters. The alignment ratio was calculated with samtools v1.6^59^. Known genes were aligned with the Core Eukaryotic Genes Mapping Approach v2.4.010312^60^ and the Benchmarking Universal Single-Copy Orthologs (BUSCO) v1.22^61^. Core Eukaryotic Genes Mapping Approach was performed using 248 highly conserved proteins and BUSCO used 4584 orthologous genes from zebrafish to evaluate the completeness of the genome assembly.

### Pseudo-chromosome assembly

We aligned previously published restriction site-associated DNA sequences from two *L. crocea* parents and 125 offspring^1^ to the improved genome assembly using Burrows-Wheeler Aligner (mem algorithm, minimum seed length = 17)^58^. Aligned sequences were genotyped with Stacks^62^ (-S -A CP -T 30—alpha 0.01 -m 5 -b 1 -X “genotypes: -o joinmap -r 90) in JoinMap format^63^ for the CP population. Loci heterozygous in both parents, with greater than two alleles, and an identical locus were filtered. We then constructed a genetic linkage map from the 8058 significantly interdependent makers (chi-squared test, *P* < 0.05). We built 24 linkage groups using ML in JoinMap v4.1, setting the minimum LOD score to 7. Scaffolds were integrated with the genetic map using the Chromonomer v1.06 (http://catchenlab.life.illinois.edu/chromonomer/).

### Gene prediction and annotation

Gene predictions were integrated with EvidenceModeler v1.1.1^64^. We used Augustus^65^ and Fgenesh from MolQuest package v 2.4.5^66^ for ab initio predictions of potential gene structure on the soft masked genome assembly generated by RepeatMasker (v3.2.9)^67^. The protein sequences of five vertebrates (*Danio rerio*, *Gasterosteus aculeatus*, *Oryzias latipes*, *Takifugu rubripes*, and *Homo sapiens*) were aligned to the *L. crocea* assembly using genBlastG^68^ and exonerate v2.2.0^69^ to identify candidate genes. Transcripts from 45 transcriptomes of *L. crocea* were aligned to the improved genome assembly using HISTAT2 (v2.1.0)^70^ and transcript assembly was using StringTie (v1.3.2)^71^. After integration with EvidenceModeler, the overlap length of each gene’s coding region was calculated based on the predicted genes from ab initio, homology and transcript methods mentioned above, and genes having at least 50% overlap by at least one method were selected. We also retained putative genes identified by Fgenesh that had at least one protein domain with an InterPro annotation^72^. Functional information for all putative genes was retrieved by aligning the predicted protein sequences to GenBank, Swissprot, and Kyoto Encyclopedia of Genes and Genomes databases using BLAST (-p blastp -e 1e-5).

### Gene family expansion and contraction

To detect the variations in the new version of *L. crocea* genome, we compared the genomic data from *L. crocea* v1.0*, L. crocea* v2.0, *D. rerio*, *G. aculeatus* and *T. rubripes*. Proteins that were greater than 50 amino acids in size were aligned by BLAST (-p blastp -e 1e-7), and Treefam^73^ was used to construct gene families. Gene family expansion and contraction analyses were performed by café (v2.1, default parameters)^74^. A conditional *P*-value was calculated for each gene family and families with conditional *P*-value less than 0.01 were considered to have a significantly accelerated rate of expansion and contraction.

### Isolation of *L. crocea* hepcidins and bioinformatics analysis

The sequences of *Lchamp* genes were obtained from the new version of *L. crocea* genome assembly. Primers were designed based on the predicted coding sequences of hepcidin genes (Supplementary Table 13). The databases of Ensembl and GenBank were used to identify the related genes of hepcidin to initially derive the syntenic relationships of hepcidin between species. Gene locus in the genome can be detected through gene annotation information. We investigated the key word hepcidin and manually checked the flanking genes to find genomic regions of hepcidins of all species. Multiple alignments were performed using the CLUSTAL W2 program (https://www.ebi.ac.uk/Tools/msa/clustalw2/). Signal peptides and predicted processing sites were calculated using SignalP v4.1 (http://www.cbs.dtu.dk/services/SignalP/) and ProP server (http://www.cbs.dtu.dk/services/ProP/), respectively. Phylogenetic tree was constructed by the Neighbor-Joining and Minimum Evolution methods using the MEGA v6.06^75^. The *dN/dS* ratios (*ω*), was calculated using a codon-based maximum likelihood method in PAML v4^76^. In brief, for each pair of hypotheses, nested models were calculated by comparing the difference in log likelihood values to a *χ*^2^ statistic to detect signals of positive selection. The hepcidin sequences retrieved from the databases for analysis were listed in Supplementary Table 14.

### Expression of hepcidin genes in *L. crocea*

To determine the tissue expression patterns of the different *LcHamp* genes, tissues including brain, gills, heart, head kidney, intestine, liver, skin, spleen, muscle, and stomach were collected from five normal large yellow croakers. Real-time PCR was performed using gene-specific primers (Hamp1-F1 and Hamp1-R1) for *LcHamp1*. The PCR cycling conditions were 30 s at 95 °C, followed by 40 cycles at 95 °C for 5 s, 58 °C for 10 s, and 72 °C for 20 s. The relative expression of *LcHamp1* was normalized against *Lcβ-actin* using the 2^–ΔΔCT^ method^37^, and was expressed relative to *LcHamp1* expression in the brain. The relative expression abundance of each *LcHamp2* isoform in heart, head kidney, intestine, liver, muscle, and spleen was determined by counting the number of clones of each LcHamp2 isoform out of 200 clones randomly selected from tissue, using a pair of conserved primers (Hamp2-F1 and Hamp2-R1) that allowed simultaneous co-amplification of all LcHamp2 isoforms.

### Promoter activity

The promoter sequences of the *Lchamp* genes were obtained from the *L. crocea* genome database and verified with cloning and sequencing. For the promoter activity assay, the promoter region of each *Lchamp* gene was cloned into the promoter-less vectors, pEGFP-l (Clontech, USA) and pGL3-Basic (Promega, USA). The recombinant plasmids, pEGFP-LchampPs and pEGFP-l (control), were transfected into EPC cells using FuGENE HD transfection reagent. After 48 h, the transfected EPC cells were observed under fluorescence microscope (Nikon, Japan).

For the luciferase assays, 5 × 10^4^ EPC cells per well were seeded in 96-well plates (Thermo Fisher Scientific, USA), incubated overnight, and then co-transfected with 2 ng of the pRL-TK plasmid plus either 100 ng of pGL3-LchampP plasmid or 100 ng of pGL3-Basic plasmid (control) using the FuGENE HD transfection reagent (Promega, USA). After 48 h of transfection, the luciferase activity of total cell lysate was measured with a GloMax 20/20 luminometer (Promega, USA). Firefly luciferase activity was normalized to Renilla luciferase activity (pRL-TK, Promega), and the relative luciferase activity was expressed as the ratio of normalized luciferase activity in cells transfected with pGL3-LchampP plasmid to the normalized luciferase activity in control cells transfected with the pGL3-Basic plasmid. To identify the active transcription factor binding sites, deleted fragments of *Lchamp* promoters were also cloned into the pGL3-Basic plasmid and their activity was measured as above description. All data were obtained from three independent experiments with each performed in triplicate.

### Antimicrobial activity of hepcidin mature peptides

The mature peptides of LcHamp1 (HAMP1: QSHISLCRYCCNCCKNKGCGYCCRF), LcHamp2-1/4 (HAMP2-1/4: SSAAKCQFCCRCCPDMIGCGICCRF), LcHamp2-2 (HAMP2-2: SSAAKCQFCCRCCPRMSGCGVCCRF), LcHamp2-3 (HAMP2-3: GSPARCRFCCR CCPGMRGCGICCRF), and LcHamp2-5 (HAMP2-5: GSPARCRFCCRCCPRMRGCGICCRF) were synthesized, oxidized, and purified by GL Biochem (Shanghai) Ltd. (Shanghai, China). Purity of the synthesized peptides was >/95%. The synthetic peptides were reconstituted in phosphate buffered saline (PBS; pH 7.4). We tested the antimicrobial activity of synthesized peptides against the five gram-negative bacteria *A. hydrophila*, *E. coli*, *V. alginolyticus*, *V. harvryi*, and *V. parahemolyticus*, and three gram-positive bacteria *B. subtilis*, *B. amyloliquefaciens*, and *S. aureus* using liquid growth inhibition assays. The minimum inhibitory concentration was considered the lowest concentration that induced a 100% decrease in the optical density of the bacterial suspension.

### Antiviral activity of *L. crocea* hepcidins

The antiviral activity of synthetic *L. crocea* hepcidins was tested in grouper spleen cells, in which a viral infection model has already been established^50^. Grouper spleen cells were seeded onto 6-well plates (Thermo Fisher Scientific, USA) for 18 h. The grouper spleen cells were pretreated for 1 h with each synthetic Hamp peptide at a final concentration of 50 µg/mL or with PBS (as a control). After pretreatment, the cells were infected with SGIV at a multiplicity of infection of 2. At 24 h post infection, cytopathic effects in the grouper spleen cells were observed microscopically (Leica Microsystems, Germany). The expression levels of two genes encoding SGIV envelope proteins (*ORF072* and *ORF086*) were measured with real-time PCR. *E. coioides β-actin* (*Ecβ-actin*) was amplified as an internal control. The expression level of *ORF072* or *ORF086* was normalized to *Ecβ-actin* using the 2^–ΔΔCT^ method. Moreover, supernatants and infected cell lysates were collected to determine the viral titer 50% tissue culture infectious dose^50^. All data were obtained from three independent experiments with each performed in triplicate.

### Detection of intracellular iron

Six groups of macrophages (1 × 10^6^ cells per well) were seeded onto 6-well plates for 18 h. To each of five of the groups, we added one synthetic hepcidin peptide from *L. crocea*, with a final concentration of 100 ng/mL. The sixth group (control) was treated with PBS. After 2 h incubation, we measured the intracellular iron concentration of macrophages with Inductively Coupled Plasma Mass Spectrometry. The fold change was expressed as the intracellular iron concentration in macrophages treated with synthetic hepcidin peptides versus the intracellular iron concentration in the control macrophages.

### Statistical analysis

Error bars indicate the standard error of the mean of the three independent experiments. Statistical significance was assessed by two-tailed Student’s *t*-test, ^*^P < 0.05, ^**^P < 0.01.

## Electronic supplementary material

Supplementary Material

## Data Availability

Sequence data that support the findings of this study have been deposited in NCBI Short Reads Archive (SRA). SMRT sequences can be found under the accession number: SRR7496619. Transcriptomic sequences can be retrieved under the following accession numbers: SRP035897, SRP092778, SRP095312, and GSE57608. The new version of *L. crocea* assembly has been deposited at DDBJ/ENA/GenBank under the accession number JRPU02000000 and the project PRJNA245366.
