# Supplementary material for: An improved genome assembly for Larimichthys crocea reveals hepcidin gene expansion with diversified regulation and function
Source: Commun Biol. 2018 Nov 16;1:195. doi: 10.1038/s42003-018-0207-3 (PMC6240063; doi:10.1038/s42003-018-0207-3)
Supplement: Supplementary file 1 — Supplementary Material [file 42003_2018_207_MOESM1_ESM.pdf]

Supplementary Figures

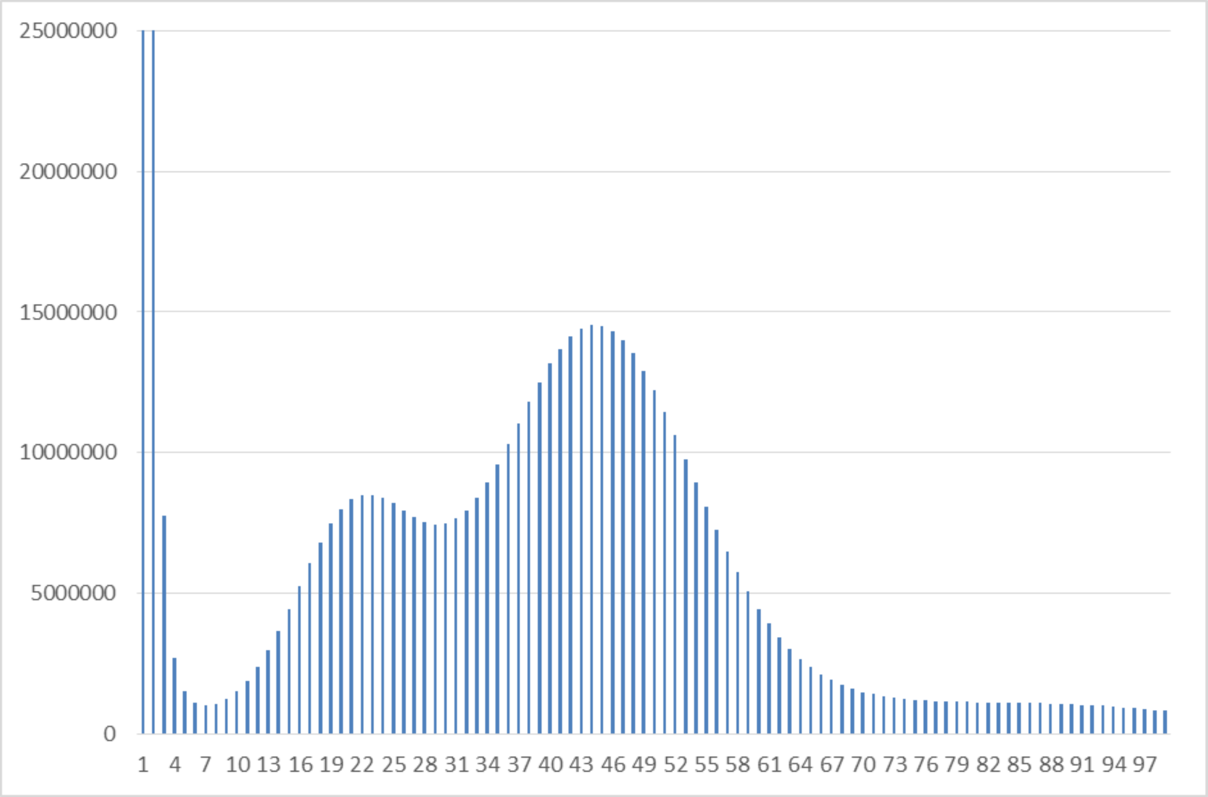

Supplementary Figure 1: K-mer analyses.

About 50-fold high quality reads were used to evaluate genome size and calculate k-mer curve by using SOAPdenovo2[1]. Jellyfish (jellyfish count -C -m 21 -s 1000000000)[2] and GenomeScope[3] were used to calculate genome Heterozygosity.

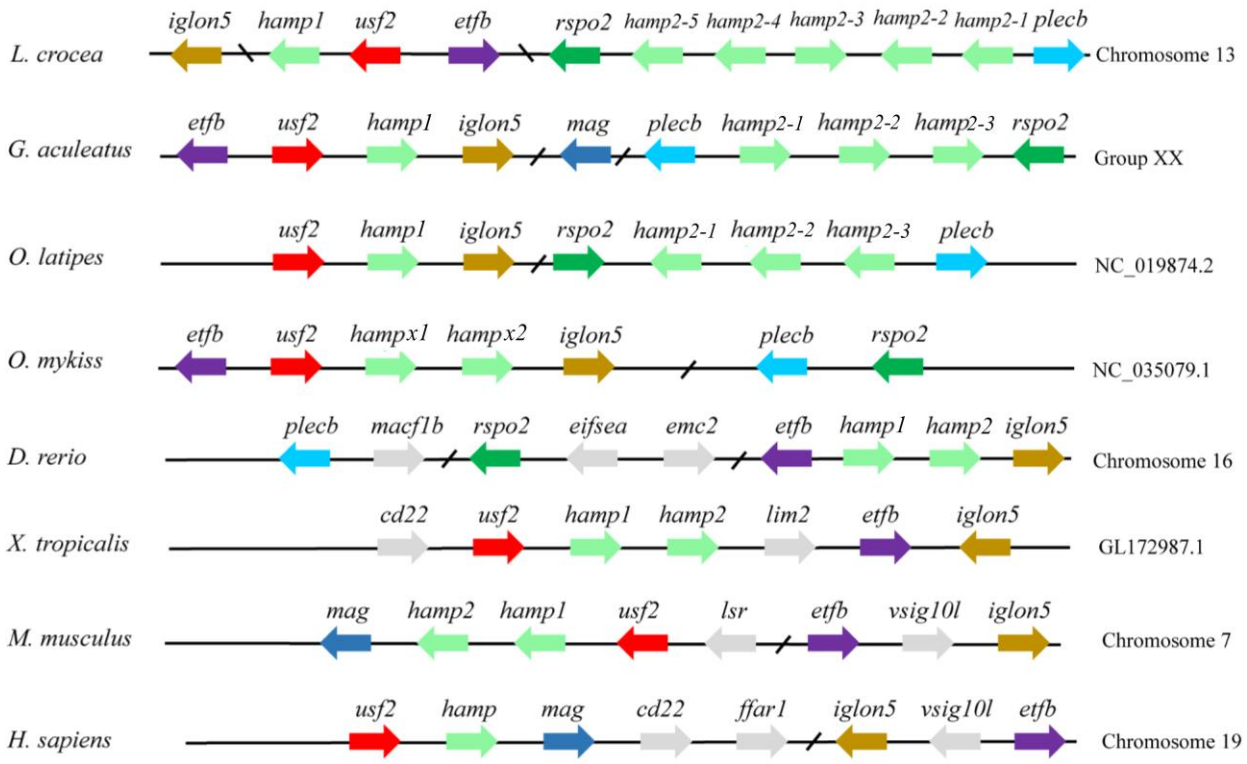

Supplementary Figure 2: Synteny of hepcidin genes between *L. crocea* and other species.

The hepcidin gene is shown in green, and the other genes are represented in gray. The conserved markers are shown in other colors. The comparison is schematic and does not reflect gene distances. Genome versions used in this analysis includes *Mus musculus* (GRCh38.p3), *Danio rerio* (GRCz10), *Mus musculus* (GRCm38.p5), *Xenopus tropicalis* (JGI 4.2), *Gasterosteus aculeatus* (BROAD S1), *Oryzias latipes* (ASM223467v1), and *Oncorhynchus mykiss* (GCA_002163495.1) in Ensembl or GenBank.

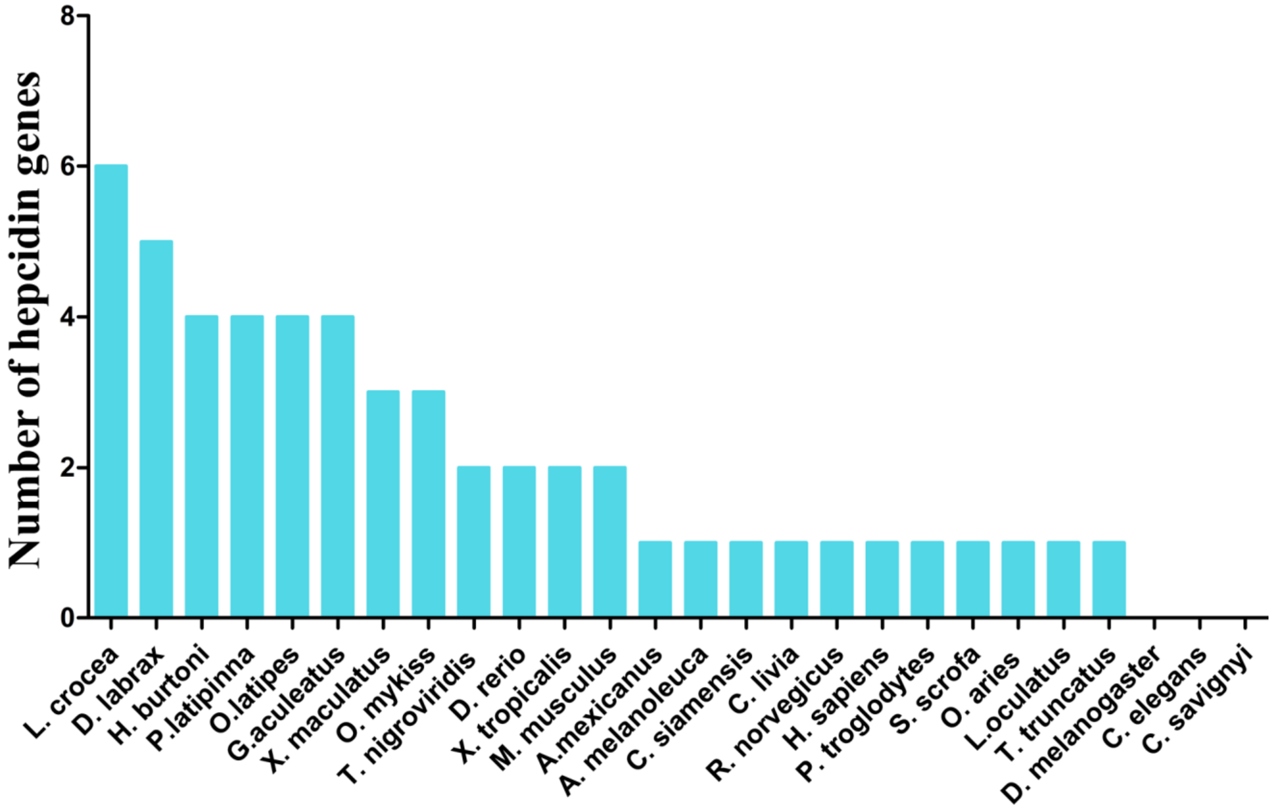

Supplementary Figure 3: The number of hepcidin genes in sequenced species.

These data were collected from Ensembl and Genbank database. The number of hepcidin genes in *L. crocea* is higher than that of other species sequenced.

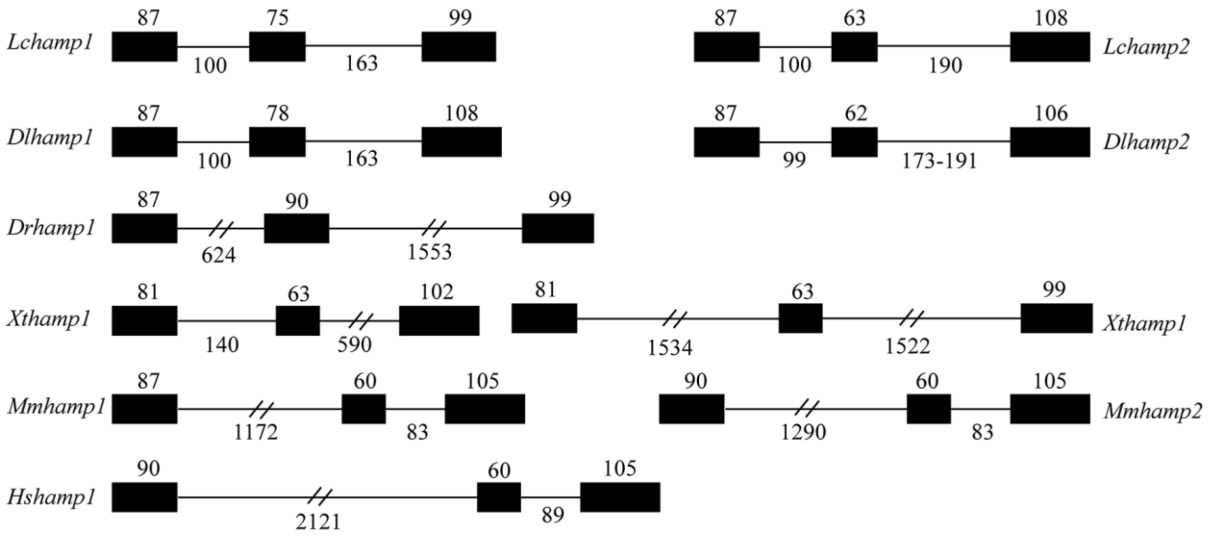

Supplementary Figure 4: Genomic structures of hepcidins from *L. crocea* and other species.

Exons are represented by boxes and their sizes in base pairs are shown on the top of the boxes. Introns are represented by a line, and the size is shown below the line. *Lc*: *Larimichthys crocea*, *Dl*: *Dicentrarchus labrax*, *Dr*: *Danio rerio*, *Xt*: *Xenopus tropicalis*, *Mm*: *Mus musculus*, *Hs*: *Homo sapiens*.

a

```
-59   ATCAGGTCTAATCTGCAAAGGATTTAATAACTAAACCATTTTTTCCAAAAAAAGCTAAA
1     ATGAAGGCATTCAGCATTGCAGTTGCAGTGACACTCGTGCTCGCCTTTATTTGCATTCTG
1      M  K  A  F  S  I  A  V  A  V  T  L  V  L  A  F  I  C  I  L

61    GAGAGCTCTGCCGTCCCATTCACCGGGGTGCAAGAGCTGGAGGAGGCAGGGAGCAATGAC
21     E  S  S  A  V  P  F  T  G  V  Q  E  L  E  E  A  G  S  N  D

121   ACTCCAGTTGCGGCACATCAAGAAACGTCAAAGGAATGGATGCCAAATTTCAGACAGAAG
41     T  P  V  A  A  H  Q  E  T  S  K  E  W  M  P  N  F  R  Q  K

181   CGCCAGAGCCACATCTCCTTGTGCCGCTACTGCTGCAACTGCTGCAAGAACAAAGGCTGC
61     R  Q  S  H  I  S  L  C  R  Y  C  C  N  C  C  K  N  K  G  C

241   GGTTACTGCTGCAGGTTCTGAGGATTCCCAGCGACAGCCAGCAAATATTAATTTATTATG
81     G  Y  C  C  R  F  *

301   CTATGCAACTTCACAGCTTTCAGGTTGTCCACTCCAAGAATTCGGGAATGCTGAATATGT
361   ATGTGCTCATCTGCAAAAACTGTACTGGTGTGCCATCCCATTAAATTTTAATGTTAGCGC
421   AAAAAAAAAATAAAAAAAAAAAAAAAA
```

b

```
-92                                  ATCAGACCAGAGAAGAACTCAGAGAGCTGACA
-61   AGACTCACCAAAAGATCTTCAGATATTTAGCTGAAGTTAAACCAGTCAAACCCTCCAAAG
1     ATGAAGACATTCAGTGTTGCAGTTGCAGTGGCCGTYRTGCTCGCCTTCATTTGTCTTCAG
1      M  K  T  F  S  V  A  V  A  V  A  V  M/V L  A  F  I  C  L  Q

61    GAGAGCTCTGCTGTCCCAGYCAATGAAGAGCAAGAGCTGGAGCAGCAMATTTATTTYGMT
21     E  S  S  A  V  P  A/V N  E  E  Q  E  L  E  Q  Q  I  Y  F  A/D

121   GATCCAGAGATGCCAGTGGAATCATGSAAGATRCCGTATYACMTGCGWSAGMAGCGTCAS
41     D  P  E  M  P  V  E  S  W/C K  M/I P  Y  H/Y L/M R  Q/E  K/Q/N  R  H/Q

181   RGCAGCSCYGCTARATGCMRGYTTTGYTGCVGWTGCTGTCSTRRWATGWBKGGATGTGGY
61     S/G S  A/P A  K/R C  Q/K/R F/L C  C  R/G C  C  P/R D/R/G M  I/S/R G  C  G

241   RTCTGCTGCAGGTTCTGA
81     I/V C  C  R  F  *
```

Supplementary Figure 5: Molecular characterization of LcHamp1 and LcHamp2.

The stop codon (TGA) is represented with an asterisk. Polymorphic nucleotides are in blue, Y: C/T, R: A/G, M: A/C, S: G/C, W: A/T, K: G/T, B: G/T/C. In the deduced amino acid sequence, the signal peptide is shaded and mature peptide is underlined. Eight conserved cysteine residues are in red.

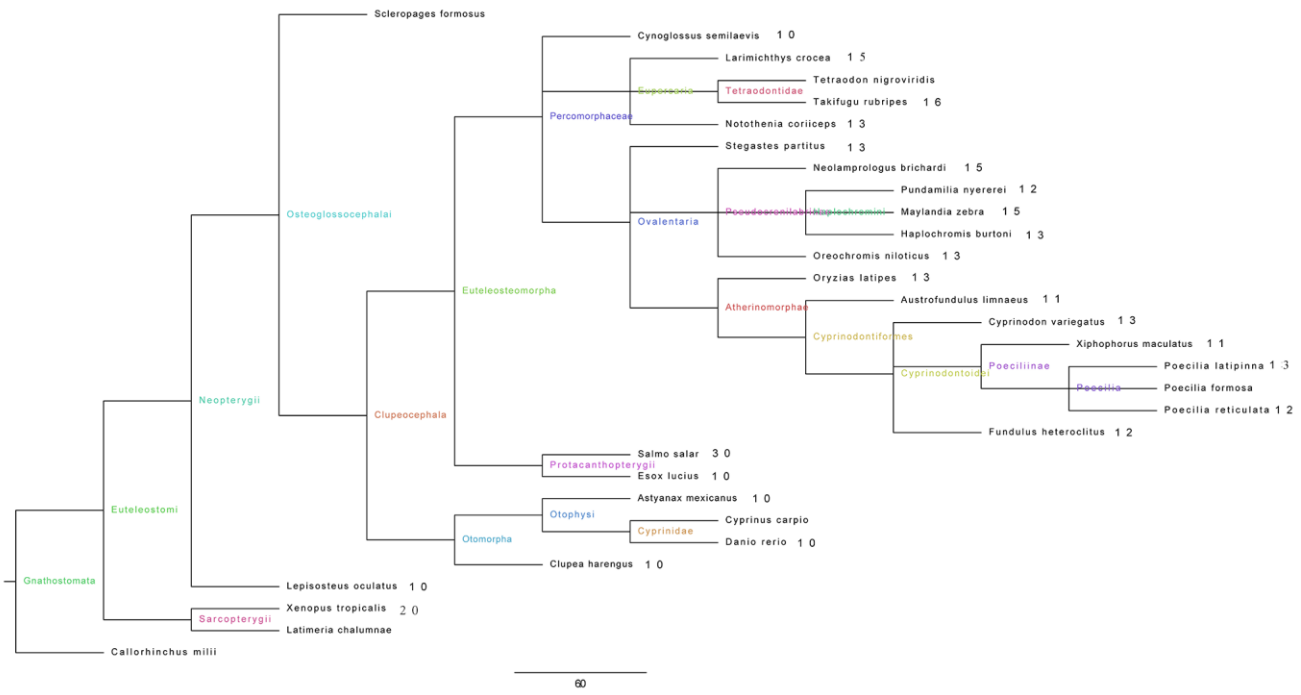

Supplementary Figure 6: Phylogenetic tree of 27 teleost fish and copy number of hepcidin genes.

The topology of the tree was obtained from NCBI taxonomy. The first number after the taxa name denotes the number of Hamp1, and the second one denotes the number of Hamp2.

a ***Lchamp1P***

```
-1690                                                     ATAAATCCCAACAAATG
                                                                 P1
-1673    TGGAGCCATCGTAGTTGTATTTGTTGACTTTTGATGTTGTGGTCACCCAAAGCAAGCAAG
-1613    CAGGCCAGCTGGGTTATCAGTGAACGAGACAGACTGACGCAGGGGTCCCGCGGCATGTTG
-1553    TTTCACTAATTGAGTCAGCCCGGGCAATTTGGATAGACTTTGAAGATATAATGAGAATTC
-1493    CAAATGGCCCATGATTTTATCAGCATGCTCAGTCCACCGTTTATCAGTTTGTACCTTTAT
-1433    TTTGCCTTTTCTGTTATTTCATTTCCACCATTATTGATTTTCGGATTTATTCACAGCCTG
-1373    ATGTTGTTCTCGAATTGTTTTCCTACTCTGACAGATTAATTAATCTTAAAGTGTTTATCG
-1313    CCGCTTCACTGATAAGAGGAAAAATAATCCTCACAATCTTGAGTAGAGTTCTTGGGTTTT
-1253    TTTTCTATGCACATGTAACAATCTGGATTATTATCATTATGTAACTGTCATTACATTCAC
-1193    ACGGTTGTAATTTCACACTGTTAATATATTTATGAACCTAACATGCTGTGTGGCAGGAAA
-1133    AATTAGTAATGATATTTTAATGAAACAAGCAGCTGTCCTTTGTCTGGATTTCAATATATC
-1073    ATTCTTACATAATGAAGAACCTAACATGCACAATATAGAAAATGACAGGGAATATATATA
                                                                   AP1
-1013    TAAAGATACAAAATATATAATGATATAGGCTAGATTTGTATTGTAAAAAAAAAAACTTGG
-953     GGCAATGCACTTCTTCGCTTTCCAGACATAAACATAATCACATTACATTATATTCGTTTT
-893     TATCTAATGCATATTGAAACACCTTGACTCTGAAATGGGAGGTTTTTAATCCTGGCTACT
-833     ATTCCGCTGCTCTCAAAGGGATGTAATTTGGTTCAACTGCAGGTTCCTGTGTATTATGCA
-773     AAAACTGAATGTGTTGAAAATGAGGGGCTAATTATTCCATTTGAGAGGTTGTAATTACAT
-713     CCTTTAGACTGAGCAATGGATATTCCAAAGTGGCTCCCTCTCTCCTTAGTGAAGGATTTC
                                AP1
-653     CTGGGTCGAAGCAAGTGCAGCTCACTCATCCGTGCAATAGATTGACCCGAGCAGTCTTGG
          HNF4        NF-κB
-593     CCTTTGCTGTGGGAATCCCCAGTTTCCAGTTTGTTCCAGCAAAAGTCATATTTCTACATG
-533     ACTCTATAGAGCGCTGCCTCAATACAGGATACATATTGCTTTCTCATATATTGTAAAAAC
                                              C/EBPβ
-473     GATGTCAGCAGCAGCAGCAGCAGCTTGTTCATGTGTGGAATTATGTGTCCTGTCTGCGGA
-413     AAGTAATTTAATTTAAAAGTTCATAGTTGATTTGATATTGTTGAACAGACGGTGGAGCCC
              P2
-353     ACAATCAAAAATAGATCAGAGAATTTCCACCAGTGTTGCAGCATTTAAAAAAAAAAAAAA
                                                                 STAT3
-293     AAGAGGAGAAAATGAGGAGGTCAAAATTTCCCCAGTGGAGTTAGGTAACTGCTGCCAGGA
                NF-κB (p50)                                 TATA box
-233     AGGGGTTGGGCCTCCCGGAGTGATGAGGCAACACTGAGCTCAAGTGTGTATAAATACCAG
                   P3        P4
-173     AACACTCTGCATGCTCAACCATCAGACAGCAGGAAGGAGTTGACAAGGGTCACCAAAAGA
-113     TCTGAAGAAATCCTCTTGACTAGACGATCACCATCCATCACTGGAGCTGAAAAAATAAAT
                                                                +1
-53      TGAAGATATTGTGGTGCTCTTTGGTGGCCTGACACCCATGAGAAAAAAGACCCATCAGGT
8        CTAATCTGCAAAGGATTTAATAACTAAACCATTTTTTCCAAAAAAAGCTAAAATG
```

b ***Lchamp2-1P***

```
-1344   CCTAGAGTAGGCTCTGAGCTGCAGGCAAAACTGAAGATCTCTGCTAGAGCATGAGTGAGA
                P1
-1284   AGCAACTCTATTGTCTTAGTTAGCTTTGCGAGAAGGTGGTTGAGCCTACAAAAACACAAC
                                                     P2
-1224   CAATAACTTGAAACTGGTGATATGAGGCACTGAATTTGATTTATACTTACTCGGAGGGGA
-1164   GAAATACAAACCCAACCAGATATATGTGTGTTTTATTGTTGTAAATAATCCACCAGCACA
            AP1
-1104   GTGATTCACTTCAACATATACACTATGGCTCAGGAAATAAATATTATGTGTCATGTTTGT
          C/EBPβ
-1044   TTCACATACTTTTCATGAGGGATTGGTGGTCATATAATGCTCCCTGAAAAAAAAAAGCAA
-984    GTAAAATGACATGGTGACTTTGTGACTGAACAGATCAAATAGTGGTTGAAAGATAAAAAT
-924    TATGGGCTCATTCCAACAGATTAGGAGCCATTTGTGGTTGTTGACACTGACATTGTGGTC
-864    ACCTGAAGCAAACACATGGGCTAGCAGGATTATCAAATAATCAAATTGAGTGAAATAGGG
-804    GATTCTGGTTGTTGCACTGGATATTTGAAACGACTGAGGTCTTTCAAATATGAGTCGTAA
-744    TTATACAGTATGTACCAAAAAGATGCTTGGAGTATGGAGTAAAAGGCTGTATGAATTATA
-684    CTTCTGCTACATTATATGTCAAGATCATAAAATTGTGATCAAACAGAGACATGTTGTTGT
                                                         P3
-624    ATTGTTGAGACAGTGCAATTAAGTAGTGCCTTTAAACCTTGCCAACTTTTCTGCTGCTCT
-564    CAAGATTAATTTGGTGTAACTGTAGGTTTCTGTGTATTATGCAAAAAAACTGAAATTGTT
-504    GAAAATTATGACTAATTATTACATTTCAGTGCTTGGAATATCATTATTTAGTCCACTTTG
                                                         HNF4
-444    GAGGTTGTGGTTTTCTAATGGGTCTGAAAAGTACAGTCATCCATGAACCTTGACCTGTGA
-384    AGACATTTTGACCTGTTTGTCTCAGGTTCAAGTTCTTTATGTATAACTACATAATTACAT
-324    AAGTTCATCCTCTGCACTCCACCATAAATCATAATTAATTATGACTTATTATTTACTCAT
-264    CCATTGATGGAATTTTTGTTTTGTTAATTGCTTAAAAGAGACCCCAACAAGCATTTTGTT
-204    GCACAACCCTGTGTTATAAAATGATAGATTGCCTGTTGTGGAACAGATAATATGGCCTAA
                                                     NFκB
-144    GGTCAAAAACAAGAAATTCCACATTCCCATTCCCAGCATCAGGGAAAATCCAGAACTCAT
          STAT5A                                              P4   TATA box
-84     TCTTGGAAGCAGGTACATTGCGCTGCTGATGAGGCAACAGAGTCCACATGAGTATAAATG
                P5                 +1
-24     TCAGCATGTTTTACACATCCAACCATCAGACCAGAGAAGAACTCAGAGAGCTGACAAGAC
37      TCACCAAAAGATCTTCAGATATTTAGCTGAAGTTAAACCAGTCAAACCCTCCAAAGATG
```

c ***Lchamp2-2P***

```
          C/EBPβ
-890  AGTATTTTGCAACTGTCATTGTGAAGTACGCCTTTATCCATAATGGTTACATTGTGAAAT
            P1
-830  GCACACGAACGGGAGAAAGAATCATGCATTGGTGTCATATCACATGGTGCTTCTTGTCAG
-770  CATATCCACAGCCGATTTTAACAGGGACTATTGTGTTGTTACTGTCTAGTTTAAGTGTGA
-710  CAAGCATCACAGAGTTTCCTGGAGAAACTGTGTGGATACAATGCTTATAAAGGTACAACA
-650  GTGTGAACTTCAGTTTCATTAACAATAAGATGACTGTACAGGTGATTTGGGAGAATATGG
-590  AGAAGAAAACATTTATGGGTATTTGGGATGCTAGAGGTAAAGACTAGAGGTGAAAGACAT
-530  TTACCATCATTCATCCCTTGTATTTGAATGCAATATGGTGACAGGACCAGAAAGATAGAA
-470  TTAATAATACCACTGCTTATATTAATCACACTGAAAATTACCATTAAATTGAAATAATTT
-410  AAATTCCATGATGGAGCCAGTATAGGAACTATTGGTAAGTTTATGTCAATCTCAAATACT
                         IRF4                             STAT6
-350  TTCAATAAGGGCTGCAATTATGTTTTCTTTATCGAAGTACTTGTGTAGTACGTTCAATGG
                     P2                  P3
-290  AAGATTTCCTATATAGTTCTATAGGAAATGTGGCTTAATCATCCATGCAATAGCCGGACC
                                                         C/EBPβ
-230  TTTAAGATGTTTGTCCTGTTCTCCCAGTAATATGCAGCAATATTAATTTGCTGTGGAATG
                    C/EBPα                 NFκB
-170  TGGAGCAAACAATATTGCACAAGGTCAAGAACAGAAAATTCCCCAGTCCTGTTCCTAACA
                                 STAT3
-110  TCATTCAGGGGATGATCTCACAACCATTCCTGGAAGCAGGTATTCCGACAGTGATGAGGT
                       TATA box                            +1
-50   AACAATGGCCCAAGCAAGTATAAATATCAGTATGCTTCACAAACCCAACCATTAGTCTGA
11    CACAGGTAACTCAAAAACACTGAGAAGAGTCATCAAAAGTCCATAAAAAATCGAGCTGCT
71    ATCTGCCTAAACCCTCCAAAGATG
```

d ***Lchamp2-3P***

```
                                                                C/EBPβ
-765   ACTGACATCAGCTAACGTCATATAGTATCACATCTGAATTTTTGCATATTGCGGCTTTCA
              P1
-705   AAACATTCTTTATATTGTCTGCACTTCAATACTCGGAGATATACAATAGTGAAACAATCT
                STAT                                         AP1
-645   ATGGATAGTTCATGGAAGAATTAATGTGACATATTTTGAACACATGAAGTGATTCATTGT
                                       P2
-585   ATGTATCCAACAAAATCAGACATTAAAAAACATTTGAAGTGGAGAAATTATACTTCATAA
-525   ACATGAAAACAAGAATATACGCCATCAACAATCTAAATAATTTTGATTAAAATACAGCTG
-465   ATCTCCATGCAGCATTTTATCAACACTGAGGCCACATGAGGTGATGTTAAATAAGGGGAA
            P3
-405   AATGAATGATGCTGACTGAAGCAGTTTTTTATTATTAATTTAAACATGGTGGATTTTTAG
                                             C/EBPβ
-345   TGTCTAAATTGTCTGTTTAGTGTACTAAACCATGTGTGCAAGGACATAAACTGATATCTA
-285   TTTTTTTGACTATGCAGAGAAAACTAGGGTATAATATAACAATATATAATTTGTTTAAAT
-225   GTTTATGTCACTGAAAACCAAAAGAATACCAAGTTATGACAGACCCTAACCCAAAAATCC
                                          NFκB
-165   TACAATCAAAAATAAACATCTCATTCTTCAGGACTTTCCTAAAACCATTCCCAGCATCAC
                              STAT3
-105   CCCTGGAGTGATGCAAGTTCATTCCTGGAAGCAGGTGTATTCTGCGCTGATGAGGCAACA
                     TATA box     P4                P5      +1
-45    GAGTCTACATGAGTATAAATGTCAGCATGTTTTACACATCCAAGCATCAGACCAGAGAAG
16     AACTCAGAGAGCTGACAAGACTCACCAAAAGATCTTCAGATATTTAGCTGAAGTTAAACC
76     AGTCAAACCCTCCAAAGATG
```

e ***Lchamp2-4P***

```
                               C/EBPβ
-955   ATCAGTTTATGTCCTTGCACACATGGTTTAGTACACTAAACAGACAATTTAGACACTAAA
                 P1
-895   AATCCACCATGTTTAAATTAATAATAAAAAACTGCTTCAGTCAGCATCATTCATTTTCCC
-835   CTTATTTAACATCACCTCATGTGGCCTCAGTGTTGATAAAATGCTGCATGGAGATCAGCT
-775   GTATTTTAATCAAAATTATTTAGATTGTTGATGGCGTATATTCTTGTTTTCATGTTTATG
-715   AAGTATAATTTTTCCACTTCAAATGTTTTTTAATGTCTGATTTTGTTGGATACATACAAT
          AP1                                    STAT5A
-655   GAATCACTTCATGTGTTCAAAATATGTCACATTAATTCTTCCATGAACTATCCATAGATT
                                          P2                    C/EBPβ
-595   GTTTCACTATTGTATATCTCCGAGTATTGAAGTGCAGACAGTATAAAGAATGTTTTGAAA
-535   GCCGCAATATGACAAAAATTCAGATGTGATACTATATGACGCTGATGTCAGTCTTATCGA
-475   CAGACACATTTCTTATATGTATTGTGTACATGTAAATAAATGTTTGTATATACCAATAAA
-415   TTGGTGTATGAGTGTTTAAATCGTATATGACACCAATATCTTAATTAAAATCAGGATTAT
-355   TTTCTGCAACAGTGGTCAATAAGGAAACAAAAACTTTTATCAAATCAGCTTTCAGTAGGT
-295   GCGTCACACCATCCTGCACCTTCCTGAGAGAAGATTATTTCATGTCTCTCCCAGTAAATT
-235   TATATGGAACACATTTTTTCCAAATAAATCTCTTAAAATTGACATCAGTACAGGATACAT
                                                     NFκB
-175   TTAGCAACTTCTTCAAAATAAAGAACATCCAATTCTTCAGGACTTTCCGAAAAACCATTC
                                           STAT3
-115   CCAGCATCACCCCTGGAGTGATGCAAGTCCATTCCTGGAAGCAGGTATATTCTGCGCTGA
                                  TATA box             P3      P4      +1
-55    TGAGGCAACAGAGTCCACATGAGTATAAATGTCAGCATGTTTTACACATCCAACCATCAG
6      ACCAGAGAAGAACTCAGAGAGCTGACAAGACTCACCAAAAGATCTTCAGATATTTAGCTG
66     AAGTTAAACCAGTCAAACCCTCCAAAGATG
```

f ***Lchamp2-5P***

```
-1411   ATCAAAGGCCTTTGACAAATCTAAAAAAATTCCTATAGTGTATTCTTTATTGTCATTAGC
            C/EBPβ    P1                                        STAT
-1351   TCTGTGTAATTCATTAATGAGATGGGTAATTGCCATTAAGGTAGAATGGGATTTCCTGAA
-1291   ATCGTATTGATGATTGTATAATATTTTAATATTGTCAAGGAAACTAAACAATCTATTGTA
                  STAT3
-1231   GGCTAATTTTTCCAGGATCGTTGAAATGGGGGGTAAAATGGAGATAGGTCTATAGTTGAT
-1171   GAATATTGTAGGGTCTTCACTTTTGTGTAAAGGAATAATTTTGGCCATTTTCAGTTCTTG
-1111   TGGCACTATTCCTTTAGTGAAAGACAAATTAAATATATGTGTCAGTGGTTTGAAATATTC
-1051   TGTTCACAGACACAGACAATGGGCCTCATGTGTTAAGTTCGCAGAGATGTGGACCTCAGA
                C/EBPβ                                             P2
-991    GCAGAGACTTACACACACACAATGGTAAATGAAAACAAAAAATACTTTAATTACAAAAAG
-931    CAAAGTTACAAGTTACCATTTTTACAGTGTATACAAAATCATACACAGCACAATTTATAG
-871    AGAAATGTCTTCTCTGCATTTAACCCATCCTAACTATTAGGAGCAGAAGGCAGATGCAGA
-811    TGAGTTTCTTTGTTTGTTTTCAGTTTAAATCTGTGATTATAAGAGTGCTAACTAGTAGCT
-751    TAGCAGGCTTAATTGCTACATTAAGCAAAAACCAACCAACTGGGGTAAAACTACAAGTCT
-691    GACGTCTGTTCCATCAGTGTTTCCATCCAAACATTCCACTCAAAACACAGTATTTCCCTT
-631    GCAGCTATAGTTTGGGTTTGATGACAAATACCACACATGAAGAACAAAAATGATTACATA
                   NF-κB
-571    AAGATTGAGGGATTTTCCTAAAACAAAATAAAATCATTAGATATAATGTAGTTGTGAAGA
-511    TTGTAGTTTTAAAACATGCTAAAGGCGATGCTCTCTGTCATATTTAAAACTAGACATTTT
-451    AAAATGTGTCAGGTGTGTTTTCTAACAGATGATGAACCATTCATTTTACACTTTGTTGAC
                                                AP1    C/EBPβ
-391    ACAGACGTGTTCACGTTTATCAGTTAAGGAGCTGAGTGATGTGAAATAAGGGATTACTGT
                      C/EBPβ
-331    TATTGTATAAGACATTAGGAAAGGCTGCTGAAACGATGCTACCTGACCTTTAGTATTAAC
-271    TGCACAGTTATTTCTTCATTTAACGGTTTATTGTTTAAATGTGTGATAATGTGCAGCAAT
-211    TTCCTCATGTATGTAACTGTGTATTCTATATGCAGAGAGGAGATACTTCATTTATTTTAG
-151    CTTTAGAGGCAGACTCATAGGCTTTAAACCCATTCCTGCATCACCCTGGGAGTGATGCAA
                            HNF3
-91     GTCATTTCCTGAAGGCAAACAGGTGCTGCCACTGACGAGGAAACAGTGGTCCACATGAGT
       TATA box              P3                 +1      P4
-31     ATAAATGTCAGCATGTTTTACACATCCAACCATCAGACCAGAGAAGAACTCAGAGAGCTG
30      ACAAGACTCACCAAAAGATCTTCAGATATTTAGCTGAAGTTAAACCAGTCAAACCCTCCA
90      AAGATG
```

Supplementary Figure 7: Predicted transcription factor binding sites of *L. crocea* hepcidin promotors.

The 5'-flanking sequences of *L. crocea* hepcidin promotors was analysed by the MatInspector program. The important transcription factor binding sites were highlighted and the primers were underlined. The initiation codon (ATG) was boxed.

Supplementary Tables

Supplementary Table 1: Summary of k-mer analysis.

Based on the k-mer analysis, the genome size of *L. crocea* is calculated to be 669 Mb.

k-mer size	k-mer number	Peak depth (bp)	Genome size (bp)	Used base	Used read	Coverage (×)	Average of read length (bp)
**17**	30,809,727,404	46	669,776,683	37,219,648,900	372,196,489	56	99

Supplementary Table 2: Statistics of SMAT sequencing.

For the PacBio Sequel system, extracted DNA was sheared into 20 kb fragments and converted into 20 kb SMRT bell template libraries. Libraries were size-selected with a lower cutoff of 7 kb and sequenced on the PacBio Sequel system. In total, three SMRT cells were sequenced, producing about 2.81 Mb of reads containing 16.70 Gb.

	**Length (bp)**	**Number**
Max length (bp)	133,081	
N10	17,253	79,596
N20	14,000	188,069
N30	12,103	316,881
N40	10,762	463,472
N50	9671	627,212
N60	8408	811,416
N70	6893	1,030,090
N80	5266	1,305,958
N90	3404	1,694,192
Total	16,695,349,496	
Number (>=100 bp)		2,813,427
Number (>=2000 bp)		2,058,015
Coverage (×)	25	

Supplementary Table 3: Summary of BACs used in *L. crocea* genome project[4].

Average Length of BAC (kbp)	BAC Number	96-well Plates	Sequence Bases (Gbp)	Average per BAC (×)	Genome Depth (×)
120	42,528	443	324.73	63.63	464

Supplementary Table 4: Information of whole-genome shotgun reads[4].

Insert Size	Average Read Length (bp)	Total Data (Gb)	Sequence Depth (×)
170 bp	100	13.95	19.62
500 bp	100	22.27	31.32
2 kbp	49	18.87	26.54
5 kbp	49	4.84	6.80
10 kbp	49	6.28	8.83
20 kbp	49	3.30	4.64
40 kbp	49	0.97	1.36
Total	----	70.48	99.11

Supplementary Table 5: Statistics of final assembly.

A 25-fold coverage of the long molecule sequences from SMRT and a 563-fold coverage of short read sequences from Illumina were used for *L. crocea* genome assembly.

	Scaffold		Contig	
	**Length (bp)**	**Number**	**Length (bp)**	**Number**
Max length	20,714,152		1,954,556	
N10	15,399,244	4	928,275	58
N20	14,021,484	9	690,983	141
N30	9,142,974	15	516,292	253
N40	7,770,016	23	387,281	403
N50	6,546,199	33	282,693	609
N60	4,494,644	45	208,118	884
N70	2,300,943	66	142,439	1,270
N80	1,353,625	104	85,085	1,874
N90	516,751	182	33,654	3,097
Total length	671,891,289		669,851,494	
Number (>=100 bp)		84,792		90,002
Number (>=2000 bp)		2,194		7,141

Supplementary Table 6: Comparison of the two versions of the *Larimichthys crocea* genome assembly.

		***L. crocea* genome assembly v1**	***L. crocea* genome assembly v2**
**Contig**	**N50 (kbp)**	63.11	282.69
	**Max length (kbp)**	716.89	1954.55
	**Total length (Mbp)**	661	669
**Scaffold**	**N50 (Mbp)**	1.03	6.55
	**Max length (Mbp)**	4.91	20.71
	**Total length (Mbp)**	679	672
	**Assigned to Chromosome (%)**	62	90
**Gene**	**Number**	25,401	26,100
	**Complete open reading frame (%)**	47.02	93.66

Supplementary Table 7: CEGMA evaluation using 248 most highly-conserved Core Eukaryotic Genes.

Version		#Prots	%Completeness	#Total	Average	%Ortho
***L. crocea* v1.0**	**Complete**	244	98.39	362	1.48	32.79
	**Partial**	248	100	389	1.57	37.9
***L. crocea* v2.0**	**Complete**	248	100	340	1.37	26.61
	**Partial**	248	100	350	1.41	29.44

Supplementary Table 8: Summary of chromosome length and genetic map of *L. crocea*.

The restriction site-associated DNA sequences from two *L. crocea* parents and 125 offspring were aligned to the improved genome assembly using BWA (mem algorithm, minimum seed length = 17). 24 linkage groups were built using ML in JoinMap v4.1, setting the minimum LOD score to 7. Scaffolds were integrated with the genetic map using the Chromonomer v1.06.

Pseudo-Chromosome ID	Number of Markers	Genetic distance (cM)	Length (bp)
**chr1**	446	672.152	43,682,218
**chr2**	251	458.217	14,376,772
**chr3**	159	10574.4	52,095,323
**chr4**	48	127.677	6,444,570
**chr5**	63	297.867	5,657,075
**chr6**	449	5657.321	27,037,660
**chr7**	243	449.83	29,365,971
**chr8**	152	509.01	33,955,600
**chr9**	49	371.328	13,800,884
**chr10**	480	757.234	40,317,218
**chr11**	344	10551.59	24,268,867
**chr12**	311	10510.3	31,676,789
**chr13**	145	6096.5	49,006,925
**chr14**	81	691.043	14,218,520
**chr15**	65	5425.245	24,124,360
**chr16**	478	5591.427	23,718,729
**chr17**	317	5526.155	29,449,875
**chr18**	83	5603.863	22,283,009
**chr19**	43	403.185	13,393,637
**chr20**	249	5643.986	18,787,343
**chr21**	108	647.202	22,273,420
**chr22**	105	467.409	23,140,888
**chr23**	83	548.836	21,415,293
**chr24**	36	355.922	19,906,365

Supplementary Table 9: Gene parameter of the new version of *L. crocea* genome.

	***L. crocea* v2.0**	***L. crocea* v1.0**
**Gene number**	26,100	25,400
**Complete ORF (%)**	93.66	47.02
**Single exon gene (%)**	0.09	9.13
**Average transcript length (bp)**	11,533	13,817
**Average CDS length (bp)**	1,706	1,765
**Average exons per gene**	9.75	9.91
**Average exon length (bp)**	175	178
**Average intron length (bp)**	1,123	1,353

Supplementary Table 10: The novel gene families identified in new version of *L. crocea* genome.

	Gene name	Number
**1**	NACHT, LRR and PYD domains-containing protein 12	3
**2**	Nuclear factor 7, ovary	3
**3**	PREDICTED: CUB and sushi domain-containing protein 1	3
**4**	Homeodomain-interacting protein kinase 2	3
**5**	Zinc finger BED domain-containing protein 1	2
**6**	PREDICTED: uncharacterized protein LOC103143207 isoform X2	3
**7**	Neurotrophin receptor-interacting factor 2	6
**8**	PREDICTED: uncharacterized protein LOC106537333	3
**9**	Transposon Ty3-I Gag-Pol polyprotein	2
**10**	PREDICTED: uncharacterized protein LOC105358072	3
**11**	PREDICTED: RNA-directed DNA polymerase from mobile element jockey-like	4
**12**	uncharacterized protein LOC103357568	2
**13**	Extracellular calcium-sensing receptor, partial	2
**14**	PREDICTED: protein NLRC3-like	3
**15**	PREDICTED: protein NLRC3-like	2
**16**	PREDICTED: protein LYRIC	3
**17**	PREDICTED: uncharacterized protein LOC105920388	3
**18**	PREDICTED: uncharacterized protein LOC106945151, partial	3
**19**	Retrovirus-related Pol polyprotein from transposon 297	2
**20**	Retrotransposable element Tf2 protein type 1	3
**21**	Retrotransposon-derived protein PEG10	3
**22**	PREDICTED: uncharacterized protein LOC101167845	2
**23**	Retrotransposable element Tf2 protein type 3	8
**24**	Ovarian cancer G-protein coupled receptor 1	2
**25**	Zinc finger protein RFP	2
**26**	PREDICTED: uncharacterized protein LOC104921804	2
**27**	PREDICTED: uncharacterized protein LOC102076250	2
**28**	PREDICTED: uncharacterized protein LOC106511803	4
**29**	PREDICTED: uncharacterized protein LOC101882769	2
**30**	PREDICTED: uncharacterized protein LOC103356573	4
**31**	PREDICTED: putative nuclease HARBI1, partial	2
**32**	PREDICTED: uncharacterized protein LOC103908834	3
**33**	PREDICTED: protein NYNRIN-like	4
**34**	PREDICTED: uncharacterized protein LOC106676917	2
**35**	PREDICTED: uncharacterized protein K02A2.6-like	2
**36**	multiple C2 and transmembrane domain-containing protein 1-like isoform X1	4
**37**	unnamed protein product	3
**38**	PREDICTED: NHS-like protein 2 isoform X1	2
**39**	Alpha-2-macroglobulin	4
**40**	PREDICTED: piggyBac transposable element-derived protein 2-like	3
**41**	PREDICTED: uncharacterized protein LOC104930589	2
**42**	PREDICTED: piggyBac transposable element-derived protein 3-like	3
**43**	PREDICTED: piggyBac transposable element-derived protein 4-like, partial	4
**44**	GTPase IMAP family member 8	3

**45**	PREDICTED: C-type lectin domain family 7 member A-like isoform X1	2
**46**	PREDICTED: uncharacterized protein LOC105941089	4
**47**	reverse transcriptase	3
**48**	PREDICTED: zinc finger protein 862	3
**49**	PREDICTED: zinc finger MYM-type protein 1-like	2
**50**	PREDICTED: pogo transposable element with KRAB domain	22
**51**	PREDICTED: pogo transposable element with KRAB domain	4
**52**	Olfactory receptor 2B11, partial [	2
**53**	PREDICTED: myosin-9-like	2
**54**	Retrotransposable element Tf2 protein type 1	2
**55**	Epidermal retinol dehydrogenase 2	3
**56**	CD276 antigen	3
**57**	PREDICTED: uncharacterized protein LOC106520580	4
**58**	Retrovirus-related Pol polyprotein from transposon TNT 1-94	2
**59**	PREDICTED: neural-cadherin-like, partial	3
**60**	PREDICTED: solute carrier family 25 member 35	2
**61**	hypothetical protein EH28_00108	2
**62**	PREDICTED: zinc finger BED domain-containing protein 5-like	3
**63**	PREDICTED: astrotactin-2-like	2
**64**	Protein NLRC3	3
**65**	Arachidonate 15-lipoxygenase B	3
**66**	PREDICTED: glutamate receptor ionotropic, kainate 2, partial	2
**67**	PREDICTED: NACHT, LRR and PYD domains-containing protein 12-like	4
**68**	hypothetical protein LOTGIDRAFT_146626, partial	2
**69**	PREDICTED: neuronal PAS domain-containing protein 1, partial	2
**70**	SAM domain and HD domain-containing protein 1	2
**71**	PREDICTED: synapsin-3-like	2
**72**	Potassium channel subfamily K member 3	2
**73**	PREDICTED: ubiquitin carboxyl-terminal hydrolase 37-like	3
**74**	DNA repair and recombination protein pif1, mitochondrial	2
**75**	PREDICTED: transcription factor IIIB 90 kDa subunit-like	3
**76**	hypothetical protein EH28_04023	2
**77**	PREDICTED: uncharacterized protein KIAA1586-like	2
**78**	Betaine--homocysteine S-methyltransferase 1, partial	2
**79**	PREDICTED: uncharacterized protein LOC104953338, partial	3
**80**	unnamed protein product	3
**81**	PREDICTED: uncharacterized protein LOC106520070	2
**82**	PREDICTED: uncharacterized protein LOC106528902	6
**83**	PREDICTED: CD109 antigen	2
**84**	myosin heavy chain, partial	3
**85**	putative DNA polymerase	15
**86**	PREDICTED: probable DNA polymerase, partial	2
**87**	PREDICTED: cortexin-3-like	3
**88**	C-C chemokine 2	4
**89**	PREDICTED: trifunctional purine biosynthetic protein adenosine-3-like, partial	2
**90**	Homeodomain-interacting protein kinase 1	2
**91**	Collagen alpha-1(VI) chain	2
**92**	LReO_3	2
**93**	PREDICTED: mucin-5AC-like	2

**94**	PREDICTED: uncharacterized protein LOC103360121	2
**95**	PREDICTED: protein NLRC3-like	2
**96**	PREDICTED: plexin-A2-like	2
**97**	PREDICTED: uncharacterized protein LOC101481537 isoform X2	3
**98**	KH domain-containing, RNA-binding, signal transduction-associated protein 3	2
**99**	proliferating cell nuclear antigen, partial	2
**100**	PREDICTED: cysteinyl leukotriene receptor 1-like	2
**101**	Teneurin-1	2
**102**	Retrovirus-related Pol polyprotein from type-1 retrotransposable element	2
**103**	PREDICTED: uncharacterized protein LOC103473208	2
**104**	PREDICTED: uncharacterized protein LOC105931704	4
**105**	PREDICTED: placenta-specific gene 8 protein-like, partial	2
**106**	PREDICTED: cadherin-8-like	2
**107**	Galanin receptor type 1	2
**108**	unnamed protein product	2
**109**	PREDICTED: uncharacterized protein LOC106513546	2
**110**	PREDICTED: NAD(P)H dehydrogenase [quinone] 1-like	2
**111**	PREDICTED: vacuolar protein 8-like	2
**112**	PREDICTED: liprin-alpha-4-like	2
**113**	Transcription elongation regulator 1-like protein	2
**114**	Maternal B9.15 protein	2
**115**	uncharacterized protein LOC105924614, partial	2
**116**	Rthyrotropin-releasing hormone-degrading ectoenzyme-like	2
**117**	PREDICTED: F-box only protein 15-like	2
**118**	PREDICTED: putative GPI-anchored protein PB15E9.01c, partial	2
**119**	tigger transposable element-derived protein 1-like isoform X1	2
**120**	Gamma-glutamyltranspeptidase 1	2
**121**	LINE-1 type transposase domain-containing protein 1	2
**122**	PREDICTED: carbonic anhydrase-related protein 10	2
**123**	Mitogen-activated protein kinase kinase kinase kinase 2	2
**124**	PREDICTED: uncharacterized protein LOC106096684	3
**125**	PREDICTED: uncharacterized protein LOC106948329	2
**126**	PREDICTED: uncharacterized protein LOC106609002 isoform X2	3
**127**	PREDICTED: uncharacterized protein LOC106609002 isoform X2	2
**128**	PREDICTED: piggyBac transposable element-derived protein 4-like	2
**129**	unnamed protein product	2
**130**	hypothetical protein M91_18051	2
**131**	uncharacterized low complexity protein	2
**132**	hypothetical protein EH28_09150	2
**133**	unnamed protein product	4
**134**	Deleted in malignant brain tumors 1 protein	2
**135**	Myosin heavy chain, fast skeletal muscle	2
**136**	PREDICTED: cytochrome P450 2K1-like	2
**137**	unnamed protein product, partial	2
**138**	PREDICTED: alpha-2-macroglobulin-P-like, partial	3
**139**	PREDICTED: GTPase IMAP family member 8-like	2
**140**	Bile salt-activated lipase	2
**141**	PREDICTED: protein-arginine deiminase type-2-like	2
**142**	unnamed protein product, partial	2

Supplementary Table 11: The novel expanded gene families identified in the new version of *L. crocea* genome.

	Gene name	*L. crocea* v2.0	*L. crocea* v1.0	Fugu	Stickback	zebrafish	*P*-values
**1**	putative RNA-directed DNA polymerase from transposon X-element	56	2	1	0	0	0
**2**	caspase recruitment domain-containing protein 8-like	24	0	0	0	2	0
**3**	pogo transposable element with KRAB domain	22	0	0	0	0	0
**4**	Histone H2B 1/2	21	17	2	21	1	0.008541
**5**	PiggyBac transposable element-derived protein 4	19	3	0	8	0	0
**6**	cAMP-dependent protein kinase inhibitor beta isoform X1	18	0	0	1	0	0
**7**	UPF0317 protein	16	0	0	8	0	0.000003
**8**	putative DNA polymerase	15	0	0	0	0	0
**9**	zinc finger BED domain-containing protein 1-like	14	8	0	11	1	0.010058
**10**	Transcription factor HES-5	14	6	3	8	5	0.000261
**11**	Histone H3	13	8	13	2	1	0.000788
**12**	C-type mannose receptor 2-like	10	8	0	6	6	0.002178
**13**	SCAN domain-containing protein 3-like	10	2	1	1	0	0
**14**	Transposon TX1 hypothetical protein	9	1	0	0	0	0
**15**	Duodenase-1	9	6	0	4	1	0.000128
**16**	Retrotransposable element Tf2 protein type 3	8	0	0	0	0	0
**17**	Low choriolytic enzyme	8	7	5	4	2	0.010761
**18**	Mannose-specific lectin	6	4	2	3	0	0.002899
**19**	P2Y purinoceptor 1	6	3	3	2	1	0.002899
**20**	CUB and zona pellucida-like domain-containing protein 1	6	4	1	2	2	0.000109
**21**	general transcription factor II-I repeat domain-containing protein 2-like	6	2	1	1	0	0.000002
**22**	hepcidin	6	1	4	4	2	0.001044
**23**	Triflin	5	3	1	1	1	0.000025
**24**	Collagen alpha-1(X) chain, partial	5	2	1	1	1	0.000025
**25**	PREDICTED: desmoglein-2-like	5	4	1	2	1	0.001044
**26**	PREDICTED: Fc receptor-like protein 5	5	2	1	1	0	0.000025
**27**	NACHT, LRR and PYD domains-containing protein 12	5	4	0	1	12	0.035352
**28**	Heat shock protein 30	5	4	1	2	1	0.001044
**29**	fast skeletal myosin heavy chain isoform mMYH-11	5	3	1	3	1	0.035352
**30**	PREDICTED: myosin-7 isoform X2	5	2	0	1	3	0.001044
**31**	chymotrypsin-like elastase family member 2A	5	3	2	2	1	0.001044
**32**	Matrix-remodeling-associated protein 8	4	2	0	1	0	0.000297
**33**	SUN domain-containing protein 1	4	3	4	0	0	0.016537

**34**	RNA-directed DNA polymerase from mobile element jockey-like	4	0	0	0	0	0.000297
**35**	feline leukemia virus subgroup C receptor-related protein 2-like isoform X3	4	3	2	1	1	0.016537
**36**	sperm acrosome membrane-associated protein 4-like	4	2	0	2	1	0.016537
**37**	differentially regulated trout protein	4	1	0	0	3	0.016537
**38**	T-cell immunoglobulin and mucin domain-containing protein 4-like	4	1	2	1	1	0.016537
**39**	rhomboid-related protein 4-like	4	1	1	1	1	0.000297
**40**	C-C chemokine 2	4	0	0	0	0	0.000297
**41**	PREDICTED: protein NYNRIN-like	4	0	0	0	0	0.000297
**42**	multiple C2 and transmembrane domain-containing protein 1-like isoform X1	4	0	0	0	0	0.000297
**43**	Rho-related GTP-binding protein RhoG	4	3	1	1	1	0.000297
**44**	Alpha-2-macroglobulin	4	0	0	0	0	0.000297
**45**	piggyBac transposable element-derived protein 4-like, partial	4	0	0	0	0	0.000297
**46**	pogo transposable element with KRAB domain	4	0	0	0	0	0.000297
**47**	Sodium-dependent dopamine transporter	4	1	2	0	0	0.016537
**48**	putative G-protein coupled receptor 151	4	1	0	0	0	0.000297
**49**	H-2 class II histocompatibility antigen, A-Q alpha chain-like	4	3	0	1	2	0.016537
**50**	Transmembrane emp24 domain-containing protein 6	4	3	1	1	1	0.000297
**51**	patatin-like phospholipase domain-containing protein 2	4	3	2	1	1	0.016537
**52**	acidic mammalian chitinase-like	4	2	0	2	2	0.016537
**53**	cytochrome P450 2J6-like	4	3	2	1	1	0.016537
**54**	myelin and lymphocyte protein-like	3	0	0	0	2	0.003529
**55**	ubiquitin carboxyl-terminal hydrolase 37-like	3	0	0	0	0	0.003529
**56**	Homeodomain-interacting protein kinase 2	3	0	0	0	0	0.003529
**57**	endogenous retrovirus group K member 19 Pol protein-like	3	1	0	0	0	0.003529
**58**	Homeodomain-interacting protein	3	1	1	0	0	0.003529
**59**	olfactory receptor 4C5-like	3	1	1	0	1	0.003529
**60**	CD276 antigen	3	0	0	0	0	0.003529
**61**	Cytochrome P450 2J2	2	1	0	0	0	0.041854
**62**	CD109 antigen	2	0	0	0	0	0.041854
**63**	Cytochrome b-c1 complex subunit 10	2	1	0	0	0	0.041854
**64**	CC chemokine	2	1	0	0	1	0.041854
**65**	mucin-5AC-like	2	0	0	0	0	0.041854
**66**	cytochrome P450 2K1-like	2	0	0	0	0	0.041854
**67**	Olfactory receptor 2B11,	2	0	0	0	0	0.041854
**68**	C-C motif chemokine 5-like isoform X2	2	0	0	0	4	0.041854

Supplementary Table 12: Evidence of positive Darwinian selection from site-specific model analyses for hepcidin genes.

The *dN/dS* ratios (*ω*), was calculated using a codon-based maximum likelihood method (CODEML) in PAML v4. In brief, for each pair of hypotheses, nested models were calculated by comparing the difference in log likelihood values to a χ^2 statistic (LRT) to detect signals of positive selection.

Site model (SM)									
**Model**	**np**	**Ln L**	**Estimates of parameters**				**Model compared**	**LRT P-value**	**Positive sites**
M3	102	-2007.067388	p:	0.19756	0.57269	0.22975			[]
			ω:	0.08959	0.59819	2.00819	M0 vs. M3	0.000000000	
M0	98	-2066.503077	ω0:		0.68029				Not Allowed
M2a	101	-2013.346559	p:	0.28097	0.50677	0.21226			[]
			ω:	0.17297	1.00000	2.55509			
							M1a vs. M2a	0.000007866	
M1a	99	-2025.099581	p:	0.51170	0.48830				Not Allowed
			ω:	0.25120	1.00000				
			p0=0.77662	p=0.79349	q=0.92208				
M8	101	-2008.573482	(p1= 0.22338)	ω= 2.07012			M7 vs.M8	0.000003608	24 T 0.985*,25 A 0.934,28 P 0.993**,30 S 0.880,31 V 0.988*,32 F 0.809,33 E 0.966*,35 T 0.999**
M7	99	-2021.105867	p=	0.53736	q=	0.48464			Not Allowed

Supplementary Table 13: Primers used in this study.

Name	Sequence (5'-3')	Function
hamp1-F	ACGGACCATTCAGACTGAGC	Gene cloning
hamp1-R	TGAAGGGGCTGCAGGGACTTGCC	
hamp2-F	TGCCAGAAGGCCGTTTACAT	
hamp2-R	TGGAGGACGCGAGGTTGTCAG	
Hamp1-F1	TTGCGGCACATCAAGAAACG	Real-time PCR
Hamp1-R1	TAACCGCAGCCTTTGTTCTTG	
Hamp2-F1	AGATGCCAGTGGAATCATGGA	
Hamp2-R1	TACCACATCCCCTCATTCTAGGA	
Actin-F	GACCTGACAGACTACCTCATG	
Actin-R	AGTTGAAGGTGGTCTCGTGGA	
*Ec*β-actin-F	TACGAGCTGCCTGACGGACA	
*Ec*β-actin-R	GGCTGTGATCTCCTTTTGCA	
Hamp1P1	CCG**CTCGAG**ATTCTTACATAATGAAGAAC	Promoter activity analysis
Hamp1P2	CCG**CTCGAG**CGGAAAGTAATTTAAT	
Hamp1P3	A**CTCGAG**GGGTTGGGCCTCCC	
Hamp1P4	AG**CTCGAG**CCCGGAGTGATGAGG	
Hamp1P-R	CCC**AAGCTT**AACTGCAATGCTGAAT	
Hamp2-1P1	AA**CTCGAG**CCTAGAGTAGGCTCTGAG	
Hamp2-1P2	AA**CTCGAG**GTGGTTGAGCCTACAAAAACAC	
Hamp2-1P3	AA**CTCGAG**TCAAACAGAGACATGTTGTTG	
Hamp2-1P4	AA**CTCGAG**TCAGGGAAAATCCAGAACTC	
Hamp2-1P5	AA**CTCGAG**TCTTGGAAGCAGGTACATTG	
Hamp2-2P1	CC**CTCGAG**AGTATTTTGCAACTGTCA	
Hamp2-2P2	CC**CTCGAG**GGCTGCAATTATGTTTTCT	
Hamp2-2P3	CC**CTCGAG**TTATCGAAGTACTTGTGTAG	
Hamp2-3P1	AA**CTCGAG**ACTGACATCAGCTAACGTC	
Hamp2-3P2	AA**CTCGAG**TGTGACATATTTTGAACAC	
Hamp2-3P3	AA**CTCGAG**CAGCTGATCTCCATGCAGCA	
Hamp2-3P4	AA**CTCGAG**GTTCATTCCTGGAAGCAG	
Hamp2-3P5	AA**CTCGAG**GCAGGTATATTCTGCGCTG	
Hamp2-4P1	AA**CTCGAG**ATCAGTTTATGTCCTTGCAC	
Hamp2-4P2	AA**CTCGAG**TGTCACATTAATTCTTCCATGAAC	
Hamp2-4P3	AA**CTCGAG**ATTCCTGGAAGCAGGTATATTC	
Hamp2-4P4	AA**CTCGAG**AGGTATATTCTGCGCTGATG	
Hamp2-5P1	AA**CTCGAG**ATCAAAGGCCTTTGACAAAT	
Hamp2-5P2	AA**CTCGAG**GTGGACCTCAGAGCAGAGAC	
Hamp2-5P3	AA**CTCGAG**TGAAGGCAAACAGGTGC	
Hamp2-5P4	AA**CTCGAG**CCACTGATGAGGAAACAGTG	
Hamp2P-R	AA**AAGCTT**AACTGCAACACTGAATGTCTTCAT	

Supplementary Table 14: Hamp sequences used for multiple alignment and phylogenetic analysis.

The hepcidin sequences were collected from Genbank and Ensembl databases.

Species	Gene name	Accession number
Poecilia latipinna (*P.latipinna*)	Hamp1	XP_007553878.1
	Hamp2-1	LOC106937285
	Hamp2-2	LOC106937286
	Hamp2-3	LOC106937289
Notothenia coriiceps (*N. coriiceps*)	Hamp1	XP_010791061.1
	Hamp2-1	XP_010783265.1
	Hamp2-2	XP_010772611.1
	Hamp2-3	XP_010791080.1
Haplochromis burtoni (*H. burtoni*)	Hamp1	XP_005925578.1
	Hamp2-1	XP_005924395.1
	Hamp2-2	XP_005924390.1
	Hamp2-3	XP_005921765.1
Oryzias latipes (*O.latipes*)	Hamp1	LOC101157971
	Hamp2-1	LOC101172220
	Hamp2-2	LOC101171972
	Hamp2-3	LOC101172228
Dicentrarchus labrax (*D. labrax*)	Hamp1	AJU35239.1
	Hamp2.1	AJU35240.1
	Hamp2.2	AJU35241.1
	Hamp2.3	AJU35242.1
	Hamp2.4	AJU35243.1
Gasterosteus aculeatus (*G.aculeatus*)	Hamp1	ENSGACG00000012473.1
	Hamp2-1	ENSGACG00000005429.1
	Hamp2-2	ENSGACG00000005425.1
	Hamp2-3	ENSGACG00000005417.1
Oncorhynchus mykiss (*O. mykiss*)	HampX1	XP_021416707.1
	HampX2	XP_021439966.1
	HampX3	XP_021450828.1
Danio rerio (*D. rerio*)	Hamp1	AAR18592.1
	Hamp2	AAR18593.1
Astyanax mexicanus (*A.mexicanus*)	Hamp	XP_007239985.1
Lepisosteus oculatus (*L.oculatus*)	Hamp	XP_006641712.1
Crocodylus siamensis (*C. siamensis*)	Hamp	ADA68357.1
Columba livia (*C. livia*)	Hamp	ABV00675.1
Xenopus tropicalis (*X. tropicalis*)	Hamp1	NP_001090729.1
	Hamp2	ABL75284.1
Mus musculus (*M. musculus*)	Hamp1	NP_115930.1
	Hamp2	NP_899080.2
Tursiops truncatus (*T.truncatus*)	Hamp	ENSTTRG00000009312.1
Bos taurus (*B.taurus*)	Hamp	NP_001107980.1
Pan troglodytes (*P. troglodytes*)	Hamp	NP_001103163.1
Rattus norvegicus (*R. norvegicus*)	Hamp	NP_445921.1
Homo sapiens (H. sapiens)	Hamp	NP_066998.1

References

1. Luo R, *et al.* SOAPdenovo2: an empirically improved memory-efficient short-read de novo assembler. *GigaScience* **1**, 18 (2012).

2. Marcais G, Kingsford C. A fast, lock-free approach for efficient parallel counting of occurrences of k-mers. *Bioinformatics* **27**, 764-770 (2011).

3. Vurture GW, *et al.* GenomeScope: Fast reference-free genome profiling from short reads. *Bioinformatics*, (2017).

4. Ao J, *et al.* Genome sequencing of the perciform fish *Larimichthys crocea* provides insights into molecular and genetic mechanisms of stress adaptation. *PLoS Genet* **11**, e1005118 (2015).
